# Targeting Aquaporin-3 Attenuates Skin Inflammation in Rosacea

**DOI:** 10.7150/ijbs.86207

**Published:** 2023-10-02

**Authors:** Mengting Chen, Qinqin Peng, Zixin Tan, San Xu, Yunying Wang, Aike Wu, Wenqin Xiao, Qian Wang, Hongfu Xie, Ji Li, Wei Shi, Zhili Deng

**Affiliations:** 1 Department of Dermatology, Xiangya Hospital, Central South University, Changsha, China.; 2 Hunan Key Laboratory of Aging Biology, Xiangya Hospital, Central South University, Changsha, China.; 3 National Clinical Research Center for Geriatric Disorders, Xiangya Hospital, Central South University, Changsha, China.; 4 Hunan Binsis Biotechnology Co., Ltd, Changsha, China.

**Keywords:** Rosacea, AQP3, Skin inflammation, NF-κB signaling, Th17 cell differentiation

## Abstract

Rosacea is a common inflammatory skin disorder mediated by the dysregulation of both keratinocytes and T cells. Here, we report that aquaporin 3 (AQP3), a channel protein that mediates the transport of water/glycerol, was highly expressed in the epidermis and CD4^+^ T cells of both rosacea patients and experimental mice. Specifically, AQP3 deletion blocked the development of rosacea-like skin inflammation in model mice with LL37-induced rosacea-like disease. We also present mechanistic evidence showing that AQP3 was essential to the activation of NF-κB signaling and subsequent production of disease-characteristic chemokines in keratinocytes. Moreover, we show that AQP3 was upregulated during T cell differentiation and promotes helper T (Th) 17 differentiation possibly via the activation of STAT3 signaling. Our findings reveal that AQP3-mediated activation of NF-κB in keratinocytes and activation of STAT3 in CD4^+^ T cells acted synergistically and contributed to the inflammation in rosacea.

## Introduction

Rosacea is a chronic inflammatory skin disease initiated and aggravated by various triggers, including heat, spicy food, and sun exposure 1, 2. Although aberrant inflammatory and neurovascular conditions in the center of the face are hallmarks of rosacea 3-5, the precise mechanism underlying pathogenesis is still not clear. Recent evidence has advanced our understanding of the pathogenesis of rosacea. Multiple cell types in the skin have been demonstrated to contribute to inflammatory and neurovascular pathways, with keratinocytes and T cells considered to be the key cell determinants of chronic inflammation, telangiectasis, and neuroinflammation 6-10. It is largely assumed that keratinocytes are activated in response to various stimuli and that activated keratinocytes exacerbate inflammation by secreting proinflammatory chemokines and cytokines that recruit and activate immune cells, especially T cells, and ultimately amplify the progression of rosacea 7, 11.

Numerous studies have demonstrated that abnormal activation of NF-kB signaling results in the production and release of multiple inflammatory mediators, causing chronic inflammation 12, 13. Recent evidence has also implicated aberrant NF-κB activation in epidermal keratinocytes during the development of rosacea 14, 15. Moreover, recent studies have highlighted the importance of the T-cell response in rosacea skin lesions; this response is dominated by a Th1/Th17 cell polarization-induced immune response that is potentially induced by various cytokines and chemokines released from epidermal keratinocytes or other cell types 6. However, the underlying regulatory mechanisms of these outcomes remain unresolved.

Aquaporin 3 (AQP3), a conserved transmembrane channel, is abundantly expressed in keratinocytes and kidney collecting duct cells 16-18. However, AQP3 is also found in innate and adaptive immune cells 19. The widespread distribution of this protein suggests that it plays a significant role in controlling a variety of physiologic processes in mammals, and this hypothesis is supported by experimental models in which AQP3 is knocked out. That is, AQP3-knockout mice exhibited epidermal hydration and skin barrier abnormalities 20 and abnormal levels of concentrated urine 21, and experimental mice with intestinal illnesses were more likely to present with diarrhea and inflammation 22. Furthermore, it has been shown that NF-κB signaling in skin keratinocytes and epidermal growth factor signaling in epithelial cells are both modulated by AQP3-mediated H_2_O_2_ transport 23, 24. Additionally, it has been demonstrated that chemokine-dependent T-cell migration depends on AQP3-mediated H_2_O_2_ transport 25, although it is unclear how AQP3 influences these physiological and pathological processes.

In the present study, our results reveal that epidermal AQP3 plays a proinflammatory role in rosacea pathogenesis by activating NF-κB signaling and cells to release chemokines, which recruit CD4^+^ T cells to lesions in the skin. Moreover, T-cell-expressed AQP3 promotes Th17 cell differentiation, probably via STAT3 phosphorylation. These findings suggest that AQP3 inhibition may be a novel therapeutic strategy for rosacea.

## Materials and Methods

### Human samples

All rosacea patients and healthy subjects diagnosed by three clinicians were recruited from the outpatients at the Dermatology Department of Xiangya Hospital. Skin biopsies were collected from the central face of patients and age-matched volunteers. Written informed consent was provided by each subject. The study was designed according to the Declaration of Helsinki and approved by the Ethics Committee of Xiangya Hospital.

### Mice

Female BALB/c and C57BL/6J mice (7-8-week-old) were purchased from Slack Company (Shanghai, China). AQP3 knockout (*Aqp3*^-/-^) mouse strains were gifted from Prof. Tonghui Ma and were generated as described previously 20. The animals were housed and bred in standard specific pathogen-free conditions with a 12-hour light-dark cycle mode. All animal experiment protocols conformed to the Ethics Committee Guidelines of Xiangya Hospital.

### Reagents

The amino acid sequence of cathelicidin LL37 was LLGDFFRKSKEKIGKEFKRIVQRIKDFLRNLVPRTES, which was commercially synthesized and purified to >95% purity by Sangon Biological Technology (Shanghai, China). TNF-α was purchased from Peprotech (Rocky Hill, NJ, USA).

### LL37-induced rosacea-like model

The method of rosacea-like lesion induced by LL37 was previously described 26. Mice were shaved 24 hours before the treatment. 40 μl 320 μM LL37 peptide (dissolved in PBS) or PBS was intradermally injected into the dorsal skin of the mice twice a day for two days. The severity of skin inflammation was evaluated as previously described 27 and skin samples were collected from the euthanized mice 12 hours later after the last injection.

### Cell isolation and culture

Primary normal human keratinocytes were isolated from the human foreskin of patients who underwent circumcision as described previously 14 and cultured in CnT-07 medium (CELLnTEC, USA). HaCaT keratinocyte cell line was cultured in conditions of Dulbecco's Modified Eagle Medium (DMEM) without calcium (Gibco), 10% fetal bovine serum (FBS, Gibco), and 2mM L-glutamine (Gibco) supplemented with 100 U/mL penicillin and 100 μg/mL streptomycin (Gibco). 293FT cell line, purchased from ATCC, was grown in DMEM with calcium-containing 10% FBS and penicillin/streptomycin. All cells were kept at 37 °C in a humidified atmosphere with 5% CO_2_.

### Treatment of HaCaT keratinocytes

HaCaT cells were stimulated with LL37 (0.5, 1, 2, 4, 8μM, Sangon), Capsaicin (1μM), or TNFα (100ng/ml, PeproTech). Cells were collected at the indicated time to perform RT-qPCR, Western blot, or Immunofluorescence. All experiments were conducted at least three times.

### Lentiviral infection and stable cell line construction

The Full-length AQP3 was amplified from the HaCaT cell and cloned into the pLVX-IRES-puro backbone with a Flag-tag. AQP3 shRNA was synthesized by Biological Technology (Shanghai, China) and cloned into the pLKO.1-puro vector (MISSION TRC). The AQP3 short hairpin RNA used in this study is as follows: shRNA#1: 5'-GGGCTGTATTATGATGCAA-3' or shRNA#2: 5'-CCTTCTTGGGTGCTGGAAT-3'). Transfection experiments were carried out with the FuGENE® HD transfection reagent (Promega). 293FT cells were cotransfected with the package vectors pMD2.G, and pSPAX.2 plasmids and AQP3 overexpression or shRNA plasmids. The supernatant was collected and concentrated for further transduction. HaCaT cells were infected and refreshed with a medium containing 1 mg/ml puromycine (Selleck) 48 hours later.

### RNA extraction and quantitative real-time PCR

Total RNA of cells or skin tissues was extracted using Trizol (Invitrogen, Thermo Fisher Scientific) and cDNA was reverse transcribed with the Maxima H Minus First Strand cDNA Synthesis Kit (Thermo Fisher Scientific) according to the manufacturer's protocols. Then the cDNA was diluted in nucleic acid-free water and quantitative real-time PCR (qRT-PCR) reactions were performed with iTaq Universal SYBR Green Supermix (Bio-Rad) on a CFX Connect Real-Time PCR Detection System (Bio-Rad). The RT-qPCR cycling parameters were 95 °C for 60 s, 95 °C for 15 s, then 60 °C for 60 s for 40 cycles, and 65 °C for 5 s. Relative mRNA expression of the gene was calculated by the formula 2^-ΔΔCt^ with GAPDH used as the housekeeping gene. Primers were listed in **Supplemental Table 1**.

### Histology

Skin samples were fixed in 10% formalin and embedded in paraffin. Skin Paraffin was sectioned into 4 μm. Sections were deparaffinized in xylene, hydrated by passing through decreasing concentrations of alcohol baths and water, then stained with hematoxylin and eosin (HE), dehydrated in increasing concentrations of alcohols and finally cleared in xylene. HE staining was conducted to evaluate the inflammation infiltration of the skin. The histological analysis was imaged by an Eclipse Ni-U Upright Microscope (Nikon). 5 randomly selected fields were selected to count the inflammatory cells.

### Immunochemistry

The hydrated paraffin sections were subjected to autoclaving in citrate buffer for 15 min for antigen retrieval. Then we inactivated the Endogenous peroxidases with 3% hydrogen peroxide and washed them with PBS for 10 min three times. The slides were blocked with 5% normal goat serum for 1h and incubated with primary antibodies overnight. And the corresponding second antibodies were used. The following primary antibodies were used: anti-human AQP3 (1:100, Sigma-Aldrich). The pictures were captured by an Eclipse Ni-U Upright Microscope (Nikon). 5 randomly selected fields were selected to accomplish the quantitation analysis.

### Immunofluorescence

Skin samples were embedded in OCT (Tissue Tek) and sectioned to 8 μm. HaCaT cells with AQP3 stable overexpression or knockdown were plated in 24-well plates coated with round coverslips and stimulated with 100ng/ml TNF-α (Cat #300-01, PeproTech) in the presence of calcium. The slides and cells were fixed in 4% formalin for 10 min and washed with PBS 3 times for 10 min. Then we blocked with 5% normal donkey serum in PBS supplemented with 0.3% Triton-X100 for 1 h and incubated with primary antibodies at 4 °C overnight. The next day the appropriate fluorophore-conjugated secondary antibodies were used and 4'-6-diamidino-2-phenylindole (DAPI) was used for nuclei visualization. Following primary antibodies were used: anti-human AQP3 (1:100, Sigma-Aldrich), anti-mouse AQP3 (1:500, Abcam), Rat anti-CD4 (1:100, eBioscience), Rabbit anti-p-p65 (1:200, Cell Signaling), Rabbit anti-p65 (1:100, Cell Signaling). The images were acquired using an Eclipse Ni-U Upright Microscope (Nikon) and 5 randomly selected fields were used to count the positive cells. The number of CD4-positive or nuclear p65-positive cells was counted in at least 3 randomly selected microscopic fields (original magnification, 200×) in each sample to calculate the mean number.

### Immunoblotting

HaCaT cells were seeded in 6-well plates and then stimulated with TNF-α, IL-1β, IL-2, or IL-6 for 24 h. AQP3 stably overexpressed or knocked down HaCaT cells were plated in 6-well plates and treated with TNF-α for 15 min. The cells were harvested and lysed in RIPA buffer (Thermo Scientific Scientific) added with protease inhibitor cocktail (Thermo Scientific Scientific). The protein concentration was measured by BCA assay (Thermo Scientific Scientific), analyzed by SDS-polyacrylamide gel electrophoresis gels (SDS-PAGE), then transferred to polyvinylidene fluoride membranes (PVDF, Millipore) and blocked with 5% skimmed milk in TBST for 1 h. The membranes were incubated with different primary antibodies at 4 °C overnight and fluorophore-conjugated corresponding secondary antibodies at room temperature for 1 h. The bands were detected with a Bio-Rad imaging system (Image Lab software).

### Isolation of human naive CD4^+^ T cells and Th cells differentiation

As was described previously 28, human peripheral blood mononuclear cells (PBMCs) were separated from the peripheral blood of healthy subjects and rosacea patients by Density gradient centrifugation using Ficoll-Paque (GE Healthcare). Naive CD4^+^ T cells were isolated from PBMCs by magnetic positive selection using the human naive CD4^+^ T Cell Isolation Kit II (Miltenyi Biotec) and cultured in RPMI 1640 medium (Gibco) with 10% fetal bovine serum (FBS, Gibco). Naive CD4^+^ T cells were activated for 5 days by following antibodies and cytokines in the presence of 5μg/ml anti-CD3 (Cat #217570, Calbiochem) and 2μg/ml anti-CD28 (Cat #217669, Calbiochem). The polarizing conditions: 10μg/ml anti-IFN-γ (Cat #16-7317-85, eBioscience) and 10μg/ml anti-IL-4 (Cat #16-7048-85, eBioscience) for Th0; 10μg/ml anti-IL-4 and 10ng/ml IL-12 (Cat #200-12, PeproTech) for Th1; 10μg/ml anti-IFN-γ and 2.5ng/ml IL-4 (Cat #214-04, PeproTech) for Th2; 10μg/ml anti-IFN-γ, 10μg/ml anti-IL-4, 5ng/ml TGF-β(Cat #100-21, PeproTech), 10ng/ml IL-6 (Cat #200-06, PeproTech), 10ng/ml IL-1β (Cat #200-01, PeproTech), and 20ng/ml IL-23 (Cat #200-23, PeproTech) for Th17; 5ng/ml TGF-βand 10ng/ml IL-2 (Cat #200-02, PeproTech) for iTreg. 1 × 10^6^ naive CD4^+^ T cells were seeded in 24-well plates and the medium was refreshed on the 3rd day.

### Isolation of mouse naive CD4^+^ T cells and Th cells differentiation

The method of isolation of mouse naive CD4^+^ T cells and Th cells differentiation was reported before 28. Spleens and lymph nodes were harvested from WT or *Aqp3*^-/-^ mice. Naive CD4^+^ T cells were isolated from splenocytes and lymph node cells by magnetic positive selection using the naive mouse CD4^+^ T cell isolation kit II (Cat #130-104-453, Miltenyi Biotec) by the manufacturer's instructions and cultured in RPMI 1640 medium (Gibco) with 10% fetal bovine serum (FBS, Gibco) in the presence of 5ug/mL anti-CD3 (Cat #16-0031-85, eBioscience) and 2 μg/ml anti-CD28 (Cat #16-0281-85, eBioscience) antibodies. Naive CD4^+^ T cells were activated with different antibodies and cytokines over different time points under following polarizing conditions: Th0 polarizing, 10ug/ml anti-IFN-γ (Cat #16-7312-85, eBioscience), 10μg/ml anti-IL-4 (Cat #16-7041-85, eBioscience); Th1 polarizing, 10μg/ml anti-IL-4, 10ng/ml IL-12 (Cat #210-12, PeproTech); Th2 polarization, 20μg/ml anti-IFN-γ, 20ng/ml IL-4 (Cat #214-14, PeproTech); Th17 polarizing, 10μg/ml anti-IFN-γ, 10μg/ml anti-IL-4, 2ng/ml TGF-β, 30ng/ml IL-6 (Cat #216-16, PeproTech), 10ng/ml IL-1β (Ca#211-11, PeproTech), 20ng/ml IL-23 (Cat #200-23, PeproTech); iTreg polarizing, 5μg/ml anti-IFN-γ, 5μg/ml anti-IL-4, 5ng/ml TGF-β (Cat #100-21, PeproTech), 10ng/ml IL-2 (Ca#200-02, PeproTech). The cells were cultured in 24-well plates and replaced with fresh medium every 3 days.

### Flow cytometry

Cell surface markers and cytokines were determined with a FACSCanto II kit (BD Biosciences). The assay was performed according to the manufacturer's protocols. The data were analyzed with the FlowJo software (Tree Star). The following antibodies were used: FITC anti-mouse CD4 (Cat #100509, BioLegend), APC anti-mouse IL-17A (Cat #506915, BioLegend).

### RNA-sequencing

Total RNA was extracted from the skin biopsies of healthy subjects/rosacea patients. According to the standard Illumina RNA-seq instructions, the libraries were prepared. A fold change > 1 and false discovery rate (FDR) < 0.05 were set to identify the differentially expressed genes (DEGs). We then performed Kyoto Encyclopaedia of Genes and Genomes (KEGG) analysis and Gene Ontology (GO) enrichment analysis of DEGs.

### Statistical analysis

All data were analyzed with the GraphPad Prism and presented as mean ± standard error of the mean (SEM). Two-tailed unpaired Student's *t*-test was used for comparisons between the two groups. When more than two groups existed, One-way ANOVA and Tukey test were conducted to determine the pairwise differences. A *p*<0.05 was considered statistically different.

### Data availability

All data needed to assess the conclusions in this study are provided in the manuscript and/or the Supplementary Materials. Sequencing data for the mouse model have been deposited in the genome sequence archive under accession number CRA012759 (https://bigd.big.ac.cn/gsa/browse). Sequencing data for the skin lesions of rosacea patients used in this study were obtained from the genome sequence archive under accession number HRA000378 (http://bigd.big.ac.cn/gsa-human/) 14.

## Results

### AQP3 Expression is Elevated in Human and Mouse Skin of Rosacea

**Figure 1:**
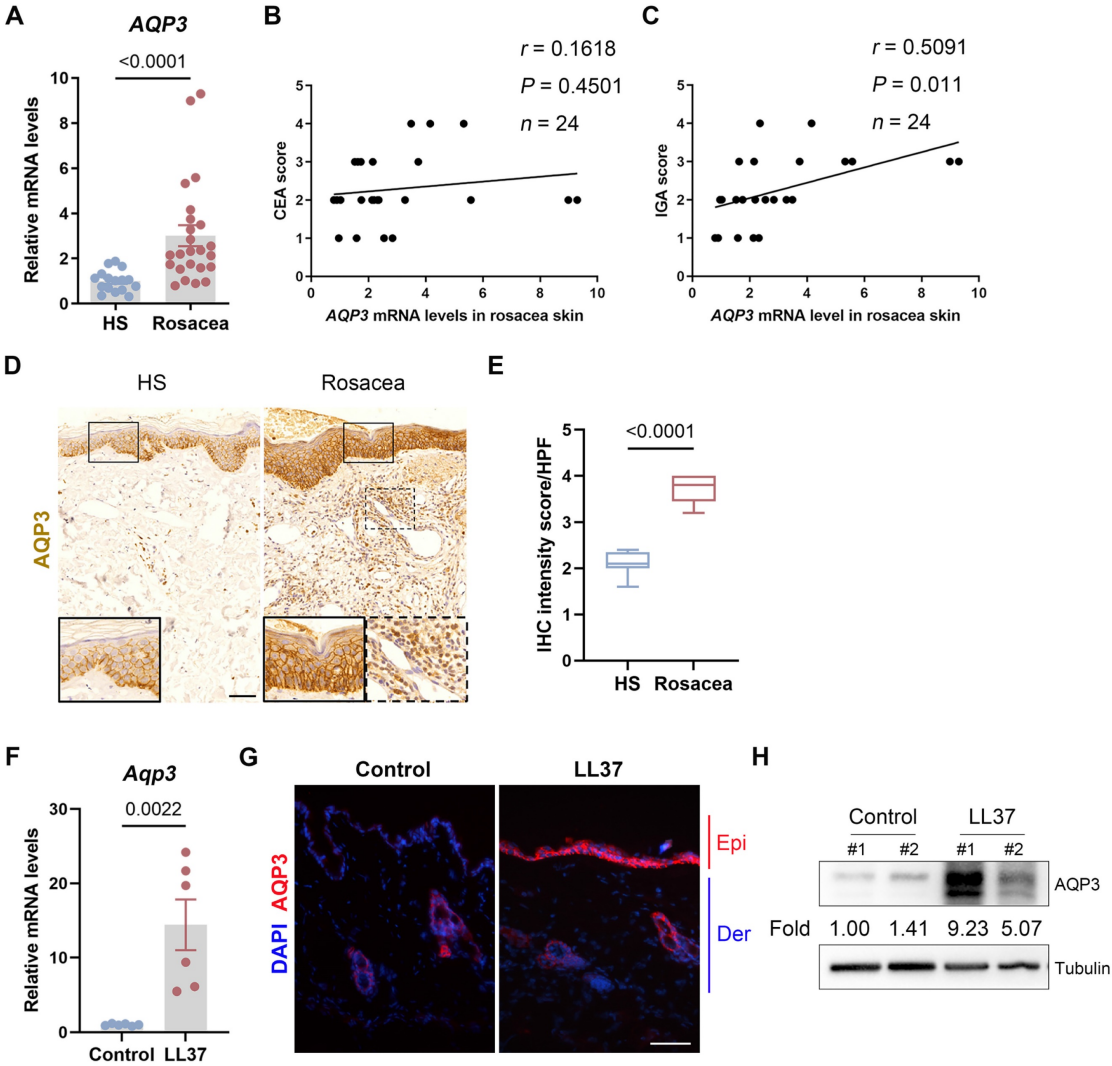
** AQP3 expression is increased in rosacea. (A)** The mRNA expression levels of *AQP3* in skin lesions from healthy individuals (n=16) and rosacea patients (n=24). HS, skin biopsies from healthy individuals; Rosacea, skin biopsies from patients with rosacea. **(B and C)** Correlation of *AQP3* mRNA levels in human rosacea skin (n=24) with the Clinician's Erythema Assessment (CEA) scores (**B**) and Clinician's Investigator's Global Assessment (IGA) scores (**C**). Spearman's correlation coefficient was used for the correlation analysis (two-tailed). **(D)** Immunohistochemistry (IHC) of AQP3 on skin sections from HS and rosacea. Higher magnified images of boxed areas are shown at the bottom of lower magnified images for each group. Scale bar: 50 μm. **(E)** The quantification of relative IHC intensity of AQP3 for each high-power field (HPF). in the epidermis in the HS and rosacea patients. Data are present the mean±SEM. Two-tailed unpaired Student's t-test was used. **(F)** The mRNA expression levels of *Aqp3* in skin lesions from Control and LL37 group mice (n = 5 for each group). Data are present the mean±SEM. Two-tailed unpaired Student's t-test was used. **(G)** Immunostaining of AQP3 in skin lesions from Control and LL37-induced mice. DAPI staining (blue) indicates nuclear localization. Epi, epidermis; Der, dermis. Scale bar: 50 μm. **(H)** Immunoblotting analysis of AQP3 in skin lysates from Control and LL37-induced mice. Tubulin is the loading control. Data are representative of at least three independent experiments.

To examine the expression and distribution of AQP3 in rosacea, we first measured the mRNA levels of *AQP3* and showed that *AQP3* expression was significantly increased in the skin lesion samples from rosacea patients compared to skin samples from healthy individuals (HS) (Figure** 1A**). Furthermore, our data showed no correlation between AQP3 expression level and Clinician Erythema Assessment (CEA) score but revealed a positive association between AQP3 expression the Investigator's Global Assessment (IGA) score (Figure** 1B-C**), which is used to assess the severity of inflammation. These results indicate that AQP3 pathogenesis is likely related to the inflammatory responses observed in rosacea. Performing immunohistochemistry (IHC), we further demonstrated that all samples from rosacea patients exhibited highly intense AQP3 staining in the epidermis and in some of the cells infiltrating the dermis, while HS showed relatively weak AQP3 activity (Figure** 1D-E**).

In addition, according to previously published studies, we established LL37-induced rosacea-like model mice, which largely manifested the clinical phenotypes and pathological changes of human disease 26, 29, 30. As described in previous studies, mouse skin treated with LL37 developed clear rosacea-like redness and histopathological changes (Figure** S1A**). Consistent with the results from studies with human patients, those from experiments with mice exposed to LL37 revealed significantly higher mRNA levels of *AQP3* in skin lesions than those in the samples obtained from control mice (Figure** 1F**). Immunofluorescence and immunoblot analysis further confirmed that AQP3 protein expression was markedly upregulated in the skin lesions of LL37-treated mice (Figure** 1G and H**). Given that LL37 upregulated AQP3 in the epidermis of rosacea model mice model, we wondered whether it induces AQP3 in keratinocytes *in vitro*. By performing immunoblotting, we confirmed that LL37 treatment increased AQP3 expression in a dose-dependent manner in keratinocytes (Figure** S1B-C**).

External stimuli, including heat, spicy food, and sun exposure, can trigger and aggravate rosacea 3, 31; therefore, we wondered whether these stimuli can lead to upregulated AQP3 protein expression in keratinocytes. To investigate the effect of these triggers on AQP3 expression in keratinocytes, we initially simulated heat by exposing cells to heat shock (37, 42, and 44°C) in a circulating water bath, and we found that heat shock significantly increased the levels of AQP3 (Figure** S1D**). In addition, we applied capsaicin and UVA radiation to simulate spicy food and sun exposure, respectively, and then measured their effects on AQP3 expression. By performing immunoblotting, we showed that these triggers markedly increased the expression of AQP3 (Figure** S1E-F**).

Taken together, these data suggest that the upregulated expression of AQP3 may be functionally involved in the inflammatory response of rosacea.

### AQP3-Knockout Mice Are Protected against Rosacea

**Figure 2:**
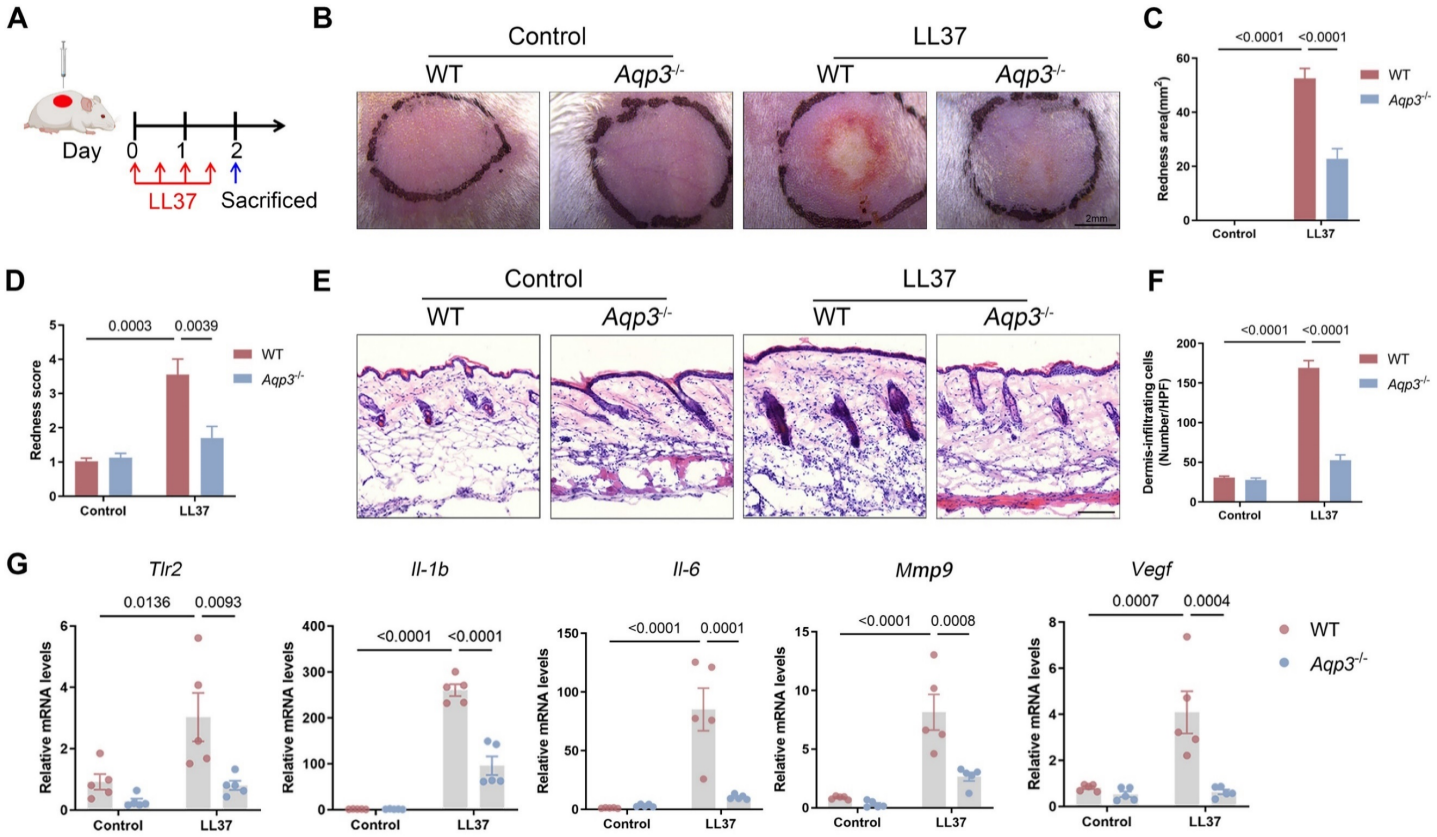
** AQP3 deficiency blocks rosacea development. (A)** Schematic diagram of intradermal injection of LL37 in mice (*Aqp3*^-/-^ and WT mice). Mice were sacrificed on day 2 to conduct subsequent experiments. The mouse experiments were repeated for three times, and 5-8 mice were included in each group for each time. The results of a representative mouse experiment were displayed. **(B)** The back skins of WT and *Aqp3*^-/-^ mice were intradermally injected with LL37 (*n*=5/group). Images were taken 48 hr after the first LL37 injection. Scale bar: 2 mm. **(C and D)** The severity of the rosacea-like phenotype was evaluated on account of the redness area **(C)** and score **(D)**. **(E)** HE staining of lesional skin sections from WT and *Aqp3*^-/-^ mice injected with LL37 (*n*=5/group). Scale bar: 50 μm. **(F)** Quantitative result of HE staining for dermal cellular infiltrates is shown. Data represent the mean ± SEM. **(G)** The mRNA expression levels of *Tlr2*, *Il-1b*, *Il-6*, *Mmp9*, and *Vegf* in skin lesions (*n*=5/group). Data represent the mean ± SEM. One-way ANOVA with Bonferroni's post hoc test was used.

To clarify the functional role of AQP3 in the development of rosacea, we established *Aqp3*-knockout (*Aqp3*^-/-^) mice. The *Aqp3*^-/-^ mice were born normally and did not show any abnormalities in weight or body size compared with age-matched wild-type (WT) mice (a population that included *Aqp3* heterozygote mice). Results from an immunofluorescence analysis confirmed that *Aqp3* was ablated in the *Aqp3*^-/-^ mice (Figure** S2A**). We injected LL37 intradermally into WT and *Aqp3*^-/-^ mice every 12 hr for a total of 4 injections (Figure** 2A**) and then compared the resulting rosacea-like phenotypes between two groups at the observation endpoint. Our results showed that WT mice exhibited typical rosacea-like dermatitis, including telangiectasia and erythema, whereas *Aqp3*^-/-^ mice did not develop obvious rosacea-like features (Figure** 2B**). AQP3 deficiency significantly reduced the redness area, and the IGA score was lower (Figure** 2C-D**). In addition, a histological analysis revealed that inflammatory cell infiltration into the dermis was significantly decreased in the *Aqp3*^-/-^ mice compared with the WT mice (Figure** 2E-F**). Moreover, an RT‒qPCR analysis showed that the expression of rosacea-related signature genes was also decreased in the *Aqp3*^-/-^ mice compared with the WT mice (Figure** 2G**). To confirm our results showing that AQP3 deficiency indeed blocks rosacea formation, AQP3-KO mice were backcrossed to mice with a C57BL/6 background, and we obtained results similar to those described above (Figure** S2B-F**). These results demonstrate that AQP3 deletion prevents pathological changes characteristic of rosacea.

### AQP3 Deficiency Suppresses NF-κB Activation in Keratinocytes

**Figure 3:**
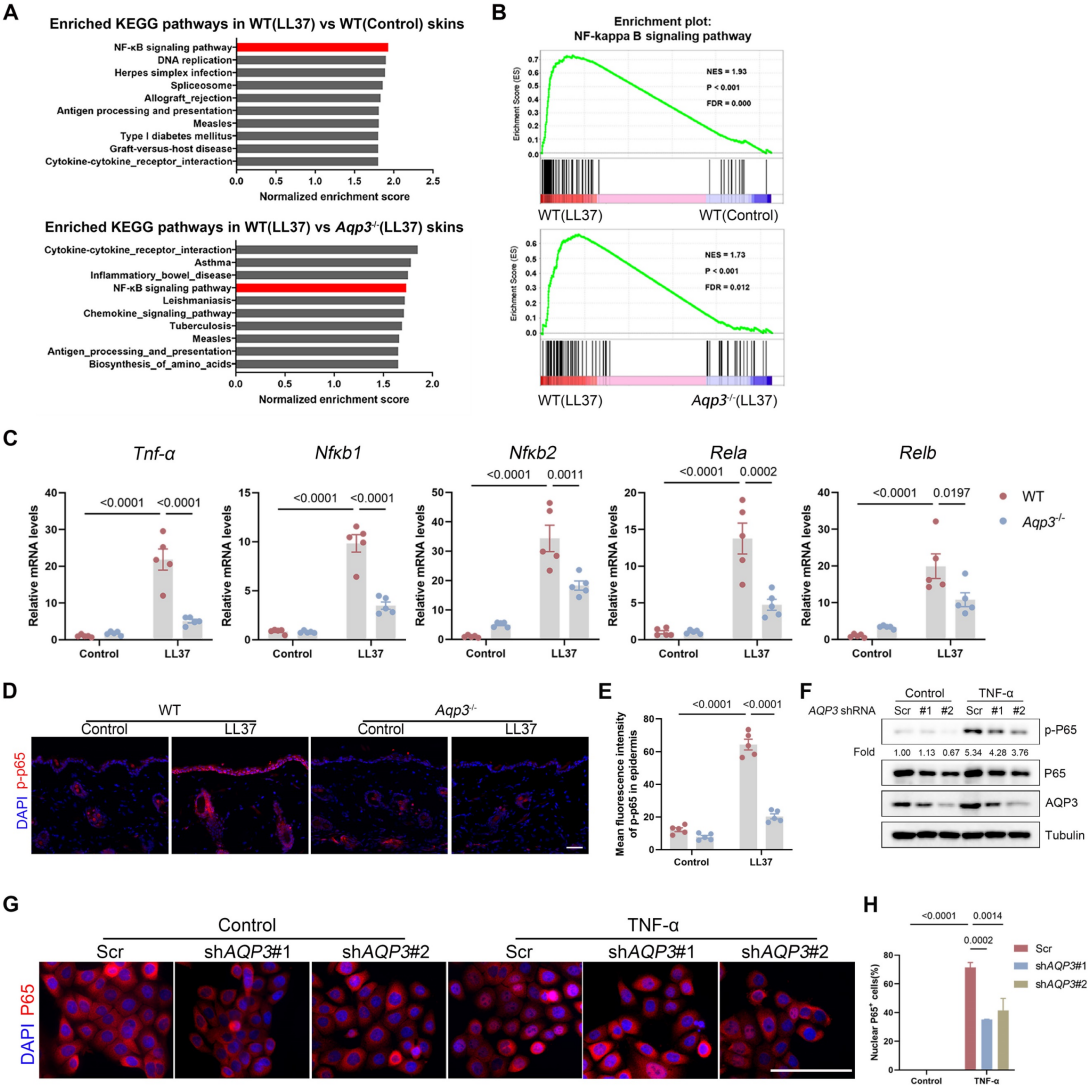
** AQP3 deficiency inhibits NF-κB activation in keratinocytes. (A)** Top-ranked enriched KEGG terms in genes that were differentially regulated respectively in the two comparisons (WT (LL37) *vs* WT (Control), and WT (LL37) *vs Aqp3*^-/-^ (LL37)) revealed by GSEA. NF-κB signaling pathway was highlighted in red box. **(B)** GSEA on RNA-sequencing data from the two comparisons both shows enrichment for NF-κB signaling pathway in LL37 group. Significance was calculated by permutation test. **(C)** The mRNA expression levels of NF-κB family of transcription factors (*Nfkb1*, *Nfkb2*, *Rela* and *Relb*) in mouse skin lesions (*n*=5/group). **(D)** Immunostaining of phospho-p65 (p-p65) in skin sections. Scale bar: 50 μm. **(E)** Quantitative result of fluorescent intensity for (D) is shown. Data represent the mean ± SEM. **(F)** Immunoblotting of p-p65 and p65 in cell lysates from scrambled and *AQP3* shRNA-transfected HaCaT keratinocytes. Cells were stimulated with TNF-α for 0.5 hr. p-p65 protein levels were analyzed relative to p65. Data are representative of at least three independent experiments. **(G)** Immunofluorescence of p65 in scrambled or *AQP3* shRNA-transfected HaCaT keratinocytes. Cells were stimulated with TNF-α for 1 hr. DAPI staining (blue) indicates nuclear localization. Scale bar: 50 μm. **(H)** Percentage of p65 positive cells in the nucleus. All results are representative of at least 3 independent experiments.

To further investigate the molecular mechanisms by which AQP3 is involved in rosacea pathogenesis, we performed RNA sequencing with skin lesions obtained from WT and *Aqp3*^-/-^ mice at baseline and after LL37 treatment. As shown in Figure **S3A**, a hierarchical clustering of genes revealed that AQP3 deficiency partly restored changes to the transcriptome induced by LL37. We identified 8207 differentially expressed genes (DEGs) between the Control and LL37-treated mice (namely, WT (LL37) *vs.* WT (Control)) and 3,635 DEGs between the WT mice and *Aqp3*^-/-^ mice treated with LL37 (WT (LL37) *vs. Aqp3*^-/-^ (LL37); *P* < 0.05 and |log2 (fold change)| > 1). Moreover, the results showed that there many of the DEGs (n=2012) were shared between any two compared groups, indicating that the ablation of AQP3 markedly attenuated molecular changes in mouse skin caused by LL37 treatment (Figure** S3A-B**). In addition, we performed KEGG pathway analysis and gene set enrichment analysis (GSEA) with each of the comparison group pairs and found that the NF-κB signaling pathway was the pathway most enriched in the WT (LL37) group, whereas NF-κB signaling pathway was not significantly enriched in group with AQP3 deficiency (Figure** 3A-B**), suggesting that this pathway might be involved in the AQP3-mediated inflammatory response in the context of rosacea.

To confirm whether AQP3 regulates NF-κB, we first measured the expression of NF-κB family of transcription factor members (namely, Nfκb1, Nfκb2, Rela, and Relb) 32, 33. By performing an RT‒qPCR analysis, we validated that the increase in the expression of these genes in LL37-induced skin was attenuated after by AQP3 deficiency (Figure** 3C**). In addition, we found that LL37 treatment increased the phosphorylation level of p65/NF-κB(p-p65) in the skin; however, the upregulated expression of p65/NF-κB(p-p65) was greatly attenuated in *Aqp3*^-/-^ mouse skin (Figure** 3D-E**). To further determine whether AQP3 modulates the NF-κB signaling pathway, we used TNF-α to induce NF-κB activation in keratinocytes *in vitro*. As described previously, TNF-α treatment significantly increased the level of p-p65, which had been decreased after AQP3 was knocked down in keratinocytes (Figure** 3F**). In addition, an immunofluorescence analysis showed that TNF-α stimulation promoted the nuclear translocation of p65, indicating the activation of NF-κB, whereas knockdown of AQP3 significantly rescued p65 transport (Figure** 3G-H**). Moreover, we measured the expression of the NF-κB family of transcription factor members and TNF-α via qPCR, and the results validated the finding that the upregulation of these genes induced by TNF-α was attenuated by AQP3 knockdown and enhanced by AQP3 overexpression (Figure** S3C-D**).

Altogether, these data suggest that AQP3 expressed in keratinocytes plays a pivotal role in skin inflammation by regulating NF-κB activation in rosacea.

### AQP3 Regulates the Production of Chemokines in Keratinocytes

**Figure 4:**
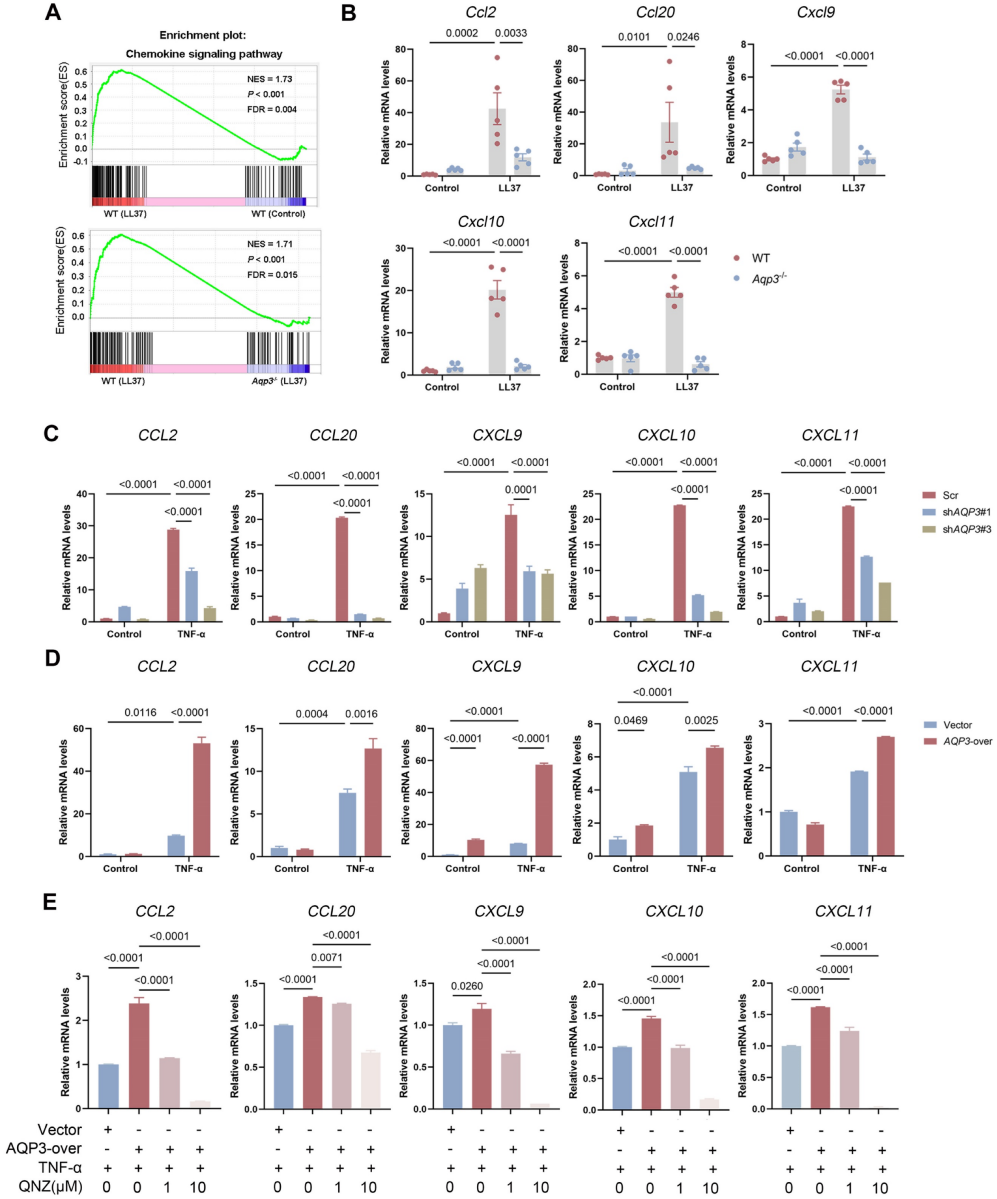
** AQP3 regulates the production of chemokines via NF-κB signaling. (A)** GSEA on RNA-sequencing data from the two comparisons (WT (LL37) vs WT (Control), and WT (LL37) vs *Aqp3*^-/-^ (LL37)) both shows enrichment for chemokine signaling pathway in LL37 group. Significance was calculated by permutation test. **(B)** The mRNA expression levels of mouse chemokines (*Ccl2, Ccl20, Cxcl9, Cxcl10, and Cxcl11*) in skin lesions (*n*=5/group).** (C)** The mRNA expression levels of chemokines in keratinocytes transfected with scrambled or *AQP3* shRNA. Data represent the mean ± SEM. One-way ANOVA with Bonferroni's post hoc test was used. **(D)** The mRNA levels of chemokines in *AQP3*-overexpressed keratinocytes treated with TNF-α. Data represent the mean ± SEM. 1-way ANOVA with Bonferroni's post hoc test was used. **(E)** The mRNA levels of chemokines in *AQP3*-overexpressed keratinocytes treated with QNZ. Data represent the mean ± SEM. 1-way ANOVA with Bonferroni's post hoc test was used.

Increasing evidence suggests that chemokines are key factors in rosacea initiation because of their ability to attract leukocytes such as T cells, neutrophils, and monocytes 34. Notably, NF-κB activation controls the production a plethora of chemokines, orchestrating the complex interactions among all components of the microenvironment in the context of disease 35. Hence, we hypothesized that AQP3 may regulate the expression of chemokines in rosacea. To verify this hypothesis, we performed a GSEA and found that the enrichment of chemokine signaling pathways was profoundly decreased in LL37-induced *Aqp3*^-/-^ mouse skin compared to WT mouse skin (Figure** 4A**). The transcriptome data were further verified via RT‒qPCR analysis. The expression of chemokines, which are crucial for recruiting T cells, was upregulated after LL37 injection; more importantly, this upregulation of chemokine expression was abolished by AQP3 deficiency (Figure** 4B**). To establish support for these results, we interfered with the expression of AQP3 in keratinocytes *in vitro*. The results showed that TNF-α-induced upregulation of chemokines was markedly attenuated by AQP3 knockdown (Figure** 4C**). In addition, AQP3 overexpression amplified the inflammation-promoting effect of TNF-α (Figure** 4D**). To investigate whether the AQP3-mediated increase in TNF-α-induced chemokines is due to the activation of NF-κB signaling, we used QNZ to inhibit NF-κB signaling. QNZ treatment significantly attenuated the expression of chemokines that had been upregulated by overexpressed AQP3 (Figure** 4E**). These data indicate that the role of AQP3 in promoting the production and release of chemokines is, at least in part, related to the NF-κB signaling activation.

### AQP3 Regulates Th17 Cell Differentiation and Promotes STAT3 Activation

Chemokines (CCL2, CCL20, CXCL9, CXCL10, CXCL11), which had been expressed after AQP3 was overexpressed, are critical mainly for the chemotaxis of CD4^+^ T lymphocytes toward keratinocytes. We hypothesized that the enhanced expression of AQP3 in keratinocytes is sufficient to recruit CD4^+^ T cells. The results from a T-cell chemotaxis assay supported our hypothesis (Figure** S4A-B**).

**Figure 5:**
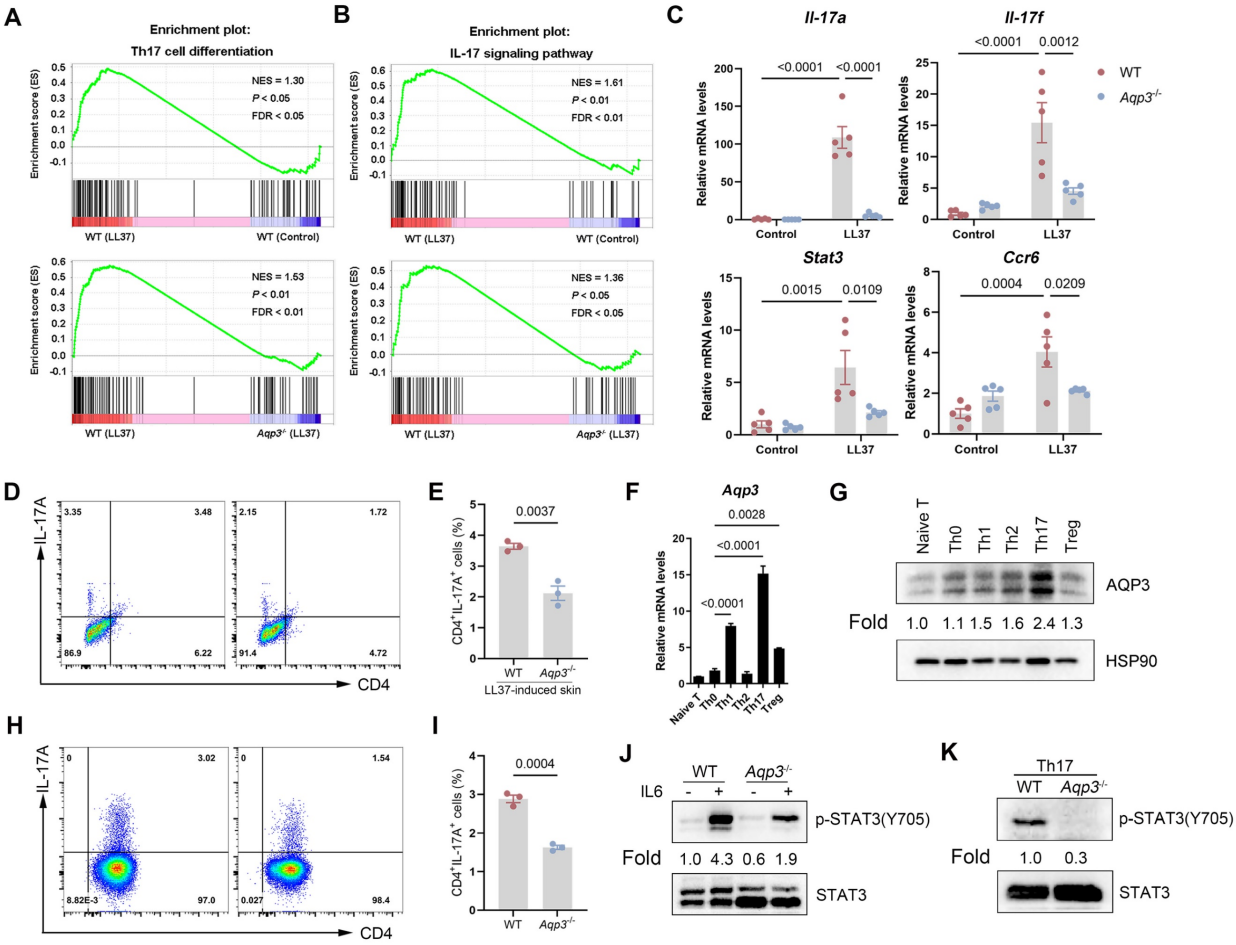
** AQP3 regulates Th17 cell differentiation and STAT3 activation. (A and B)** GSEA on RNA-sequencing data from the two comparisons (WT (LL37) vs WT (Control), and WT (LL37) vs *Aqp3*^-/-^ (LL37)) both shows enrichment for Th17 cell differentiation (**A**) and IL17-signaling pathway (**B**) in LL37 group. Significance was calculated by permutation test. **(C)** The mRNA expression levels of Th17 polarization-related genes (*Il17a*, *Il17f*, *Stat3* and* Ccr6*) in skin lesions. **(D)** Flow cytometric analysis of Th17 cells in dermal CD4^+^ T cells from WT or *Aqp3*^-/-^ mice skin treated with LL37. **(E)** Statistical analysis data of the percentage of Th17 cells in (**D**).** (F)** The mRNA expression levels of *Aqp3* in freshly isolated CD4^+^ T cells (naive) and polyclonally activated CD4 T cells (Th0) and Th1, Th2, Th17, and iTreg cells. **(G)** Immunoblotting of AQP3 in freshly isolated CD4^+^ T cells (naive) and polyclonally activated CD4^+^ T cells (Th0) and Th1, Th2, Th17, and iTreg cells. HSP90 was used as a loading control. **(H)** Mouse naive CD4^+^ T cells isolated from WT and *Aqp3*^-/-^ mice and then were differentiated into Th17 cells. The percentage of Th17 cells was detected by flow cytometry. **(I)** Statistical analysis data of the percentage of Th17 cells in (**H**). **(J)** WT or *Aqp3*-deficient naive CD4^+^ T cells were stimulated with recombinant mouse IL-6 (10 ng/ml) and collected cell lysates for immunoblot analysis. **(K)** Immunoblotting was performed to identify total and phosphorylated (Y705) levels of STAT3 in WT or *Aqp3*-deficient Th17 cells. Data represent the mean ± SEM. Two-tailed unpaired Student's t-test (**E and I**) or 1-way ANOVA with Bonferroni's post hoc test (**C and F**) was used.

As mentioned previously, we found that AQP3 is highly expressed in dermis-infiltrating cells. Through immunofluorescence assays, we found that the expression of AQP3 in CD4^+^ T cells was enhanced in rosacea patients (Figure** S5A**). Moreover, a GSEA of KEGG pathways based on RNA-sequencing data obtained from rosacea patients in our previous study suggested that multiple T-cell response-related pathways were highly enriched in the lesional skin of rosacea patients compared to those in HS (Figure** S5B**). Hence, we wondered whether the elevated expression of AQP3 in CD4+ T cells is important to rosacea pathogenesis. To test this hypothesis, we first performed GSEA. Our data showed that LL37 treatment induced a marked increase in the Th1/Th17 cell differentiation rate and in the activation of the IL-17 signaling pathway, whereas Th17 cell differentiation rate and IL-17 signaling pathway activation, but not the Th1 cell differentiation rate, were significantly repressed by *Aqp3* deletion (Figure** 5A-B**, Figure** S6A**). In addition, an RT‒qPCR analysis confirmed our results (Figure** 5C**, Figure** S6B**), suggesting that AQP3 preferentially regulates Th17 cell-polarization-induced inflammation in the context of rosacea. More importantly, we found that the percentage of Th17 cells in LL37-induced mouse skin was reduced in *Aqp3*^-/-^ mice compared with WT mice (Figure** 5D-E**). Furthermore, we isolated CD4^+^ T cells obtained from dermal cell suspensions obtained from LL37-induced rosacea-like skin samples and verified that AQP3 was elevated in CD4^+^ dermal T cells but not in CD4^-^ dermal T cells (Figure** S6C**). These findings indicate that the increased expression of AQP3 may be functionally important in the Th17 cell-mediated immune response in the context of rosacea.

To determine whether the increase in AQP3 expression contributes to the activation and differentiation of T cells in rosacea, we examined AQP3 expression in different Th cell subtypes. We induced the differentiation of mouse and human naive CD4^+^ T cells into Th1, Th2, Th17, and Treg cells under different polarization conditions *in vitro* (Figure** S6D-G**, Figure** S7A**). We found that the *Aqp3* mRNA levels were significantly upregulated in the Th1 and Th17 cells compared with the Th0 cells, while the AQP3 protein levels were markedly increased only in the Th17 cells (Figure** 5F-G**, Figure** S7B-C**). To further directly evaluate whether Th17 differentiation requires AQP3, we isolated naive CD4^+^ T cells from WT and *Aqp3*^-/-^ mice and cultured the cells under Th17-polarizing conditions *in vitro*. We performed flow cytometry to measure the percentage of CD4^+^ IL17A^+^ cells. The results showed that the rate of naive CD4^+^ T-cell differentiation toward Th17 cells in *Aqp3*^-/-^ mice was markedly lower than that in WT mice (Figure** 5H-I**). Additionally, an RT‒qPCR analysis confirmed that the expression of Th17 polarization-related genes (including *Il17a*, *Rorc*, *Stat3,* and *Ccr6*) was increased in WT Th17 cells but was significantly decreased in* Aqp3*^-/-^ Th17 cells (Figure** S6H**). Similarly, we observed that AQP3 overexpression slightly promoted the differentiation of naive CD4^+^ T cells into Th17 cells (Figure** S7D-F**). These observations confirmed that AQP3 is involved in Th17 cell differentiation.

IL-6 induces Th17 cell differentiation through the activation of STAT3 signaling 36, 37, and the phosphorylation of STAT3 at the Y705 residue is required for Th17 cell differentiation 38. To investigate how AQP3 regulates Th17 cell differentiation, we initially examined the phosphorylation status of STAT3. We found that the phosphorylation rate of STAT3 at Y705 induced by IL-6 was reduced in *Aqp3*-deficient CD4^+^ T cells compared with WT CD4^+^ T cells (Figure** 5J**). Moreover, fully differentiated *Aqp3*-deficient Th17 cells displayed markedly lower levels of phosphorylated STAT3 (Figure** 5K**).

Collectively, these data suggest that AQP3 plays an important role in regulating Th17 cell differentiation, which may be mediated by STAT3 activation in the context of rosacea.

### Targeting AQP3 Using Small Interfering RNA Protects against Inflammation in Rosacea

**Figure 6:**
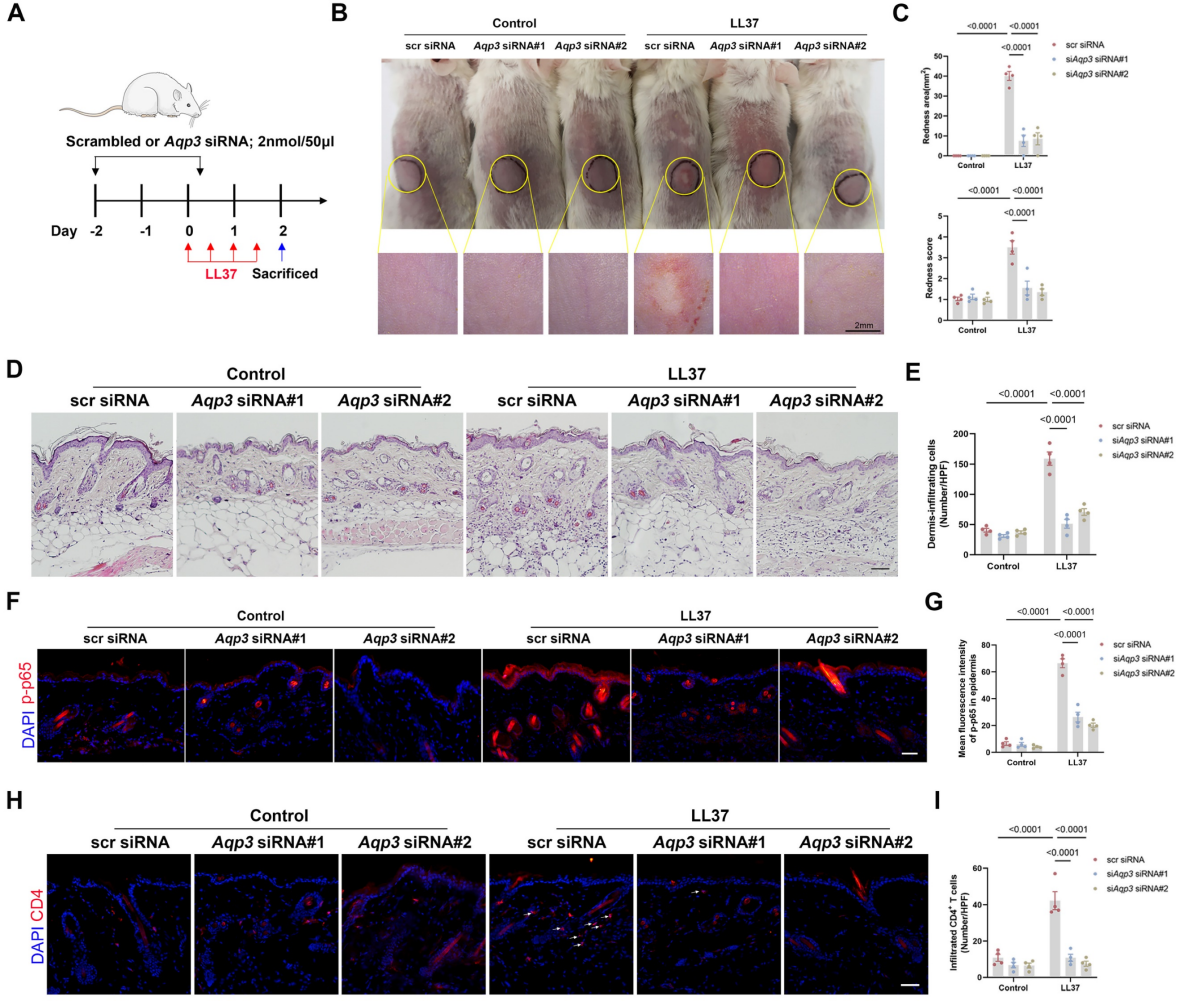
** Therapeutic targeting of AQP3 by specific siRNA protects mice from rosacea. (A)** Schematic representation of the treatment protocol with LL37 and siRNA (scrambled or AQP3). Mice were euthanized on day 2 after LL37 injection (*n*=5/group). Scale bar: 2 mm. **(B)** The back skins of mice were intradermally injected with LL37 and siRNA (scrambled or AQP3) (*n*=5/group). Images were taken 48 hr after the first LL37 injection. **(C)** The severity of the rosacea-like phenotype was evaluated on account of the redness area and score. **(D)** HE staining of lesional skin sections (*n*=5/group). Scale bar: 50 μm. **(E)** Quantitative result of HE staining for dermal cellular infiltrates is shown. Data represent the mean ± SEM. **(F)** Immunostaining of phospho-p65 (p-p65) in skin sections. Scale bar: 50 μm. **(G)** Quantitative result of fluorescent intensity for (F) is shown. Data represent the mean ± SEM. **(H)** Immunostaining of CD4 in skin sections. Scale bar: 50 μm. **(I)** Quantitative result of CD4^+^ T cells is shown. Data represent the mean ± SEM.

To further explore the possibility that AQP3 is a therapeutic target and exclude the systemic effects of AQP3 global knockout, we investigated whether silencing AQP3 in mice using small interfering RNA (siRNA) protects mice against rosacea (Figure** 6A**). *Aqp3* siRNA (the effect was validated *in vivo* by AQP3 immunostaining, Figure** S8**) was delivered to skin and found to protect mice from LL37-induced skin inflammation, as evidenced by a decrease in areas of redness and IGA score, the number of dermis-infiltrating cells, and the expression levels of disease-specific markers (Figure** 6B-E,** Figure** S9A**). The therapeutic effect of *Aqp3* siRNA was mediated via the same mechanisms identified with *Aqp3*-null mice, i.e., downregulation of NF-κB signaling (Figure** 6F-G,** Figure** S9B**), decreased expression of chemokines (Figure** S9C**), and reduced CD4^+^ T-cell infiltration and expression of polarization-related genes (Figure** 6H-I**, Figure** S9D**).

## Discussion

The present study demonstrates that AQP3 is upregulated in the epidermal keratinocytes and dermal CD4^+^ T cells of rosacea patients and model mice. In a mouse with rosacea induced by LL37 application, AQP3 knockout led to profound resistance to rosacea pathogenesis. In keratinocytes, AQP3 plays an essential role in NF-κB activation and subsequent chemokine production. In addition, we found that AQP3 deficiency inhibited Th17 cell differentiation, possibly by decreasing the level of phosphorylated STAT3. Collectively, these findings suggest that AQP3 is needed for the activation of epidermal NF-κB and chemokine signaling and reveal a previously unrecognized role for AQP3 in Th17 cell-mediated immunity in rosacea pathogenesis.

AQP3 has been extensively studied as a transporter of water, glycerol, and H_2_O_2_ in various cell types and is thus involved in the pathogenesis of multiple diseases, including psoriasis 23, 24, 39. Here, we show that AQP3 expression is increased in epidermal skin lesions from both rosacea patients and model mice. Moreover, we show a positive correlation between AQP3 and IGA score, which are used mainly to evaluate the severity of inflammation in rosacea patients. In previous studies, AQP3 was described as being closely associated with T-cell migration 24 and macrophage-dominated inflammation 39. Based on this evidence, we hypothesized that AQP3 may be implicated in the inflammation characteristic of rosacea. Indeed, in the present study, deletion of AQP3 significantly attenuated the acquisition of the inflammatory phenotype of rosacea by mice, and a subsequent transcriptome data analysis revealed the involvement of AQP3 in inflammation-related signaling pathways, such as the NF-κB and Th17 cell differentiation-related pathways.

Constitutive activation of NF-κB in keratinocytes may contribute to immune dysfunction and initiate an inflammatory cascade in patients with rosacea 14, 27. In addition, an increasing number of studies have reported that NF-κB activation leads to the production and secretion of cytokines and chemokines, contributing to inflammation through the recruitment and activation of immune cells 40-42. More importantly, these cytokines and chemokines mediate autocrine and paracrine loops among keratinocytes, as well as the crosstalk between keratinocytes and immune cells 43. Herein, we show that AQP3 positively regulates with NF-κB and the subsequent expression of chemokines such as CCL20 and CXCL9/10/11, which play important roles in amplifying the immune response in the skin lesions associated with rosacea 44, 45. Consistently, a recent study reported that AQP3-mediated intracellular H_2_O_2_ production is a central event involved in NF-κB signaling activation during the development of psoriasis 23. Therefore, we speculated that AQP3 may mediate NF-κB activation by transporting H_2_O_2_ in cells in the context of rosacea. However, the specific mechanism by which AQP3 controls these processes needs to be further explored.

In addition to keratinocytes, immune cells play a pivotal role in the pathogenesis of rosacea 7. A recent study revealed that Th1/Th17 polarization-related inflammation is a critical driver of rosacea 6. Consistently, we demonstrated that multiple T-cell-related immune response pathways were activated both in the skin lesions of rosacea patients and model mice, and naive T cells differentiated into Th1/Th17 cells in parallel with increased expression of AQP3 in CD4^+^ T cells from humans and mice with rosacea*.* This association implies a potential role for AQP3 in regulating Th1 and Th17 cell differentiation. In this context, we found that AQP3 expression was higher in differentiated Th17 cells than in other Th cell subtypes, consistent with previous sequencing data showing that AQP3 is increased during Th17 cell differentiation 46*.* Th17 cells have been identified as a distinct lineage of CD4^+^ Th cells that produce IL-17A and IL-17F, which are indispensable for autoimmune responses 36. Notably, our data suggest that AQP3 is a positive regulator of Th17 cell differentiation. These results revealed a previously unidentified role of AQP3 in T-cell differentiation, in addition to its migration- and proliferation-promoting roles. Notably, we did not find a role for AQP3 in naive T-cell differentiation into Th1 cells; thus, it will also be important to investigate why AQP3 deficiency affects only the differentiation of naive T cells into Th17 cells.

AQP3 has been shown to enhance STAT3 phosphorylation, contributing to the stemness maintenance of stem cells 47. Moreover, STAT3 is a key mediator of Th17 differentiation that integrates signaling mediated by the IL6 receptor complex 36, 37. Our results demonstrate that AQP3 deficiency significantly reduces the levels of phosphorylated STAT3 in Th17 cells, suggesting that STAT3 may mediate the regulatory effects of AQP3 on Th17 cell differentiation. However, whether the observed phosphorylation of STAT3 was due to direct phosphorylation catalyzed by AQP3 or caused by another indirect mechanism needs to be further investigated.

In the present study, in keratinocytes, we found that AQP3 positively regulates the production of chemokines (CCL2, CCL20, CXCL9, CXCL10, CXCL11), which are mainly responsible for attracting T cells 48. Among them, CCL20 is the only known high-affinity ligand that binds to CCR6 and drives CCR6^+^ cells' migration in flamed tissues (CCR6 is highly expressed in Th17 cells) 44, 49; in T cells, we found that AQP3 may promote Th17 cell differentiation via STAT3 phosphorylation. Moreover, to determine the AQP3-mediated synergistic effect between keratinocytes and CD4^+^ T cells in the pathogenesis of rosacea, we perform the t-cell chemotaxis experiment. We found that the enhanced expression of AQP3 in keratinocytes recruits more CD4^+^ T cells. Therefore, these data and references at least indirectly support our speculation that AQP3 may promote Th17 cell recruitment by regulating chemokines secreted from keratinocytes.

In summary, AQP3 functions as a proinflammatory regulator in keratinocytes and CD4^+^ T cells of rosacea skin. AQP3-dependent activation of NF-κB and subsequent release of chemokines from keratinocytes promotes CD4^+^ T cells recruitment; meanwhile, AQP3-dependent activation of STAT3 may induce CD4^+^ T cells into pathogenic Th17 cells. These events act synergistically and contribute to the inflammation in rosacea. AQP3 may be envisaged as, to our knowledge, a previously unreported therapeutic target for rosacea.

## Supplementary Material

Supplementary figures and table.

## Abbreviations

AQP3: aquaporin 3
Th1: helper T (Th) 1
Th17: helper T (Th) 17
H_2_O_2_: hydrogen peroxide
WT: wild-type
KO: knockout
IGA: Investigator's Global Assessment
CEA: Clinician's Erythema Assessment
IHC: immunohistochemistry
HS: healthy individuals
DEGs: differentially expressed genes
IL: interleukin
CCL: C-C motif chemokine ligand
CXCL: C-X-C motif chemokine ligand
