# Supplementary material for: Targeting Aquaporin-3 Attenuates Skin Inflammation in Rosacea
Source: Int J Biol Sci. 2023 Oct 2;19(16):5160–73. doi: 10.7150/ijbs.86207 (PMC10620828; doi:10.7150/ijbs.86207)
Supplement: Supplementary file 1 — Supplementary figures and table. [file ijbsv19p5160s1.pdf]

Supplemental Figures

- **Figure S1. AQP3 is induced by rosacea trigger factors.**
- **Figure S2. AQP3 deletion alleviates rosacea-like features in mice**
- **Figure S3. AQP3 positively regulates NF-κB activation in keratinocyte.**
- **Figure S4. The enhanced expression of AQP3 in keratinocyte promotes $CD4^+$ T cell chemotaxis in the presence of TNF-α.**
- **Figure S5. AQP3 is also upregulated in $CD4^+$ T cell.**
- **Figure S6. AQP3 regulates Th17-polarization inflammation.**
- **Figure S7. AQP3 involves in human Th17 cell differentiation.**
- **Figure S8. Validation of the knockdown effect of Aqp3 siRNA in the skin.**
- **Figure S9. Deficiency of Aqp3 suppresses rosacea development**

Supplemental Table

- Supplemental Table 1: List of primers used for qRT-PCR.

Supplemental Figures

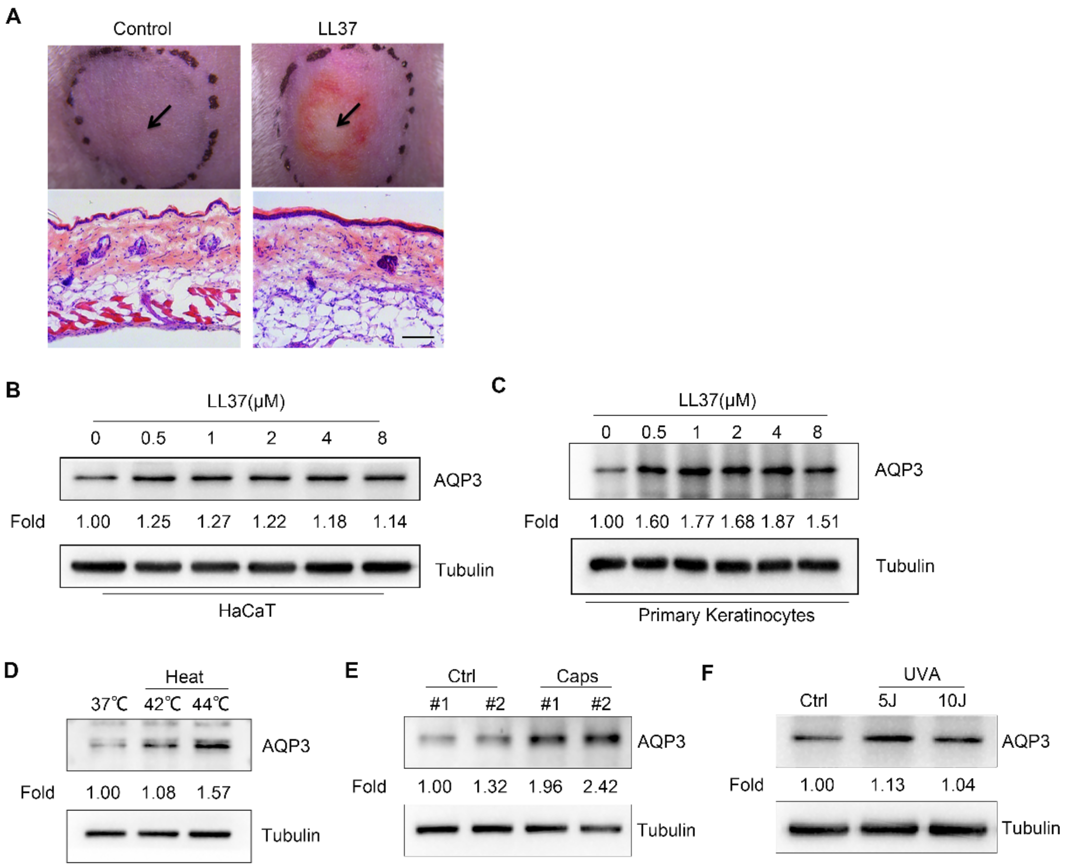

Figure S1. AQP3 is induced by rosacea trigger factors.

(A) Representative images of rosacea-like skin inflammation in mice at baseline and following LL37 injection. Scale bar: 50μM

(B) Immunoblotting of AQP3 in cell lysates from HaCaT keratinocytes treated with different doses of LL37 (0-8 μM) for 24 hr. Tubulin is the loading control.

(C) Immunoblotting of AQP3 in cell lysates from primary human keratinocytes treated with different doses of LL37 (0-8 μM) for 24 hr. Tubulin is the loading control.

(D) Immunoblotting analysis of AQP3 in cell lysates from HaCaT keratinocytes stimulated with heat shock (37-44℃) for 24 hr.

(E) Immunoblotting analysis of AQP3 in cell lysates from HaCaT keratinocytes stimulated with capsaicine (1 μM) for 24 hr.

(F) Immunoblotting analysis of AQP3 in cell lysates from HaCaT keratinocytes

treated with UVA. Data are representative of at least three independent experiments.

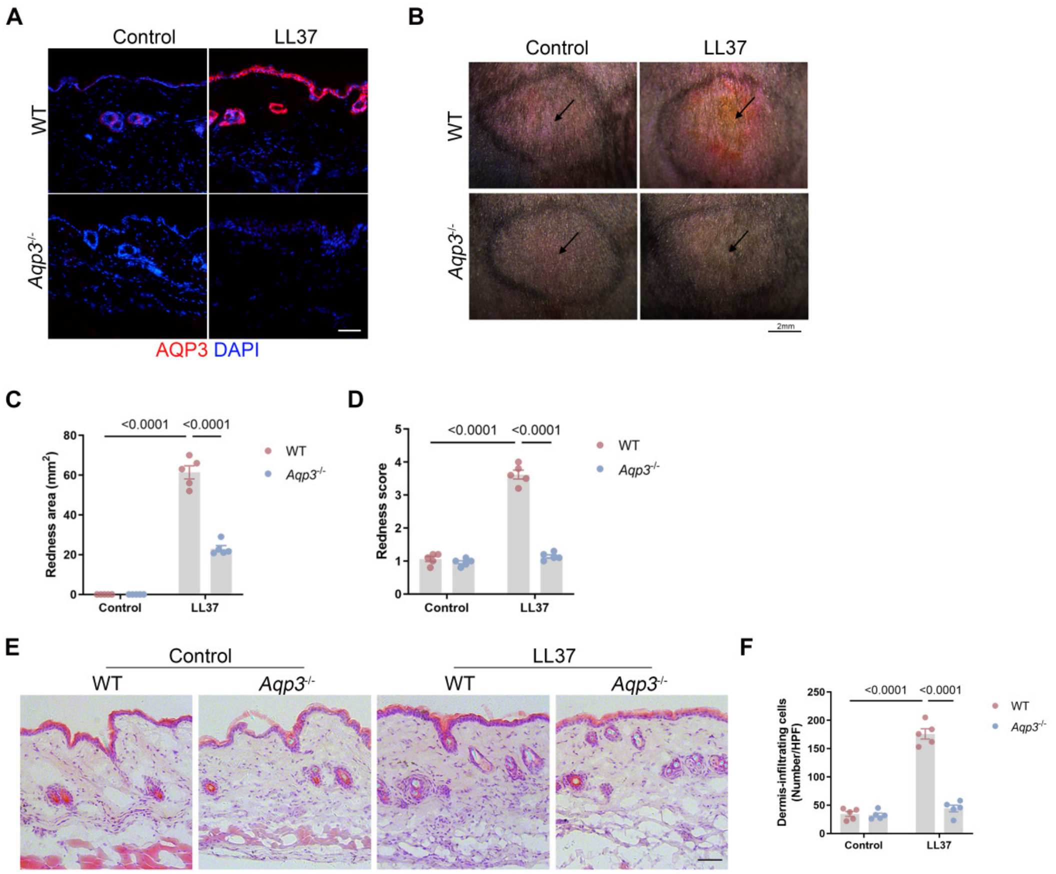

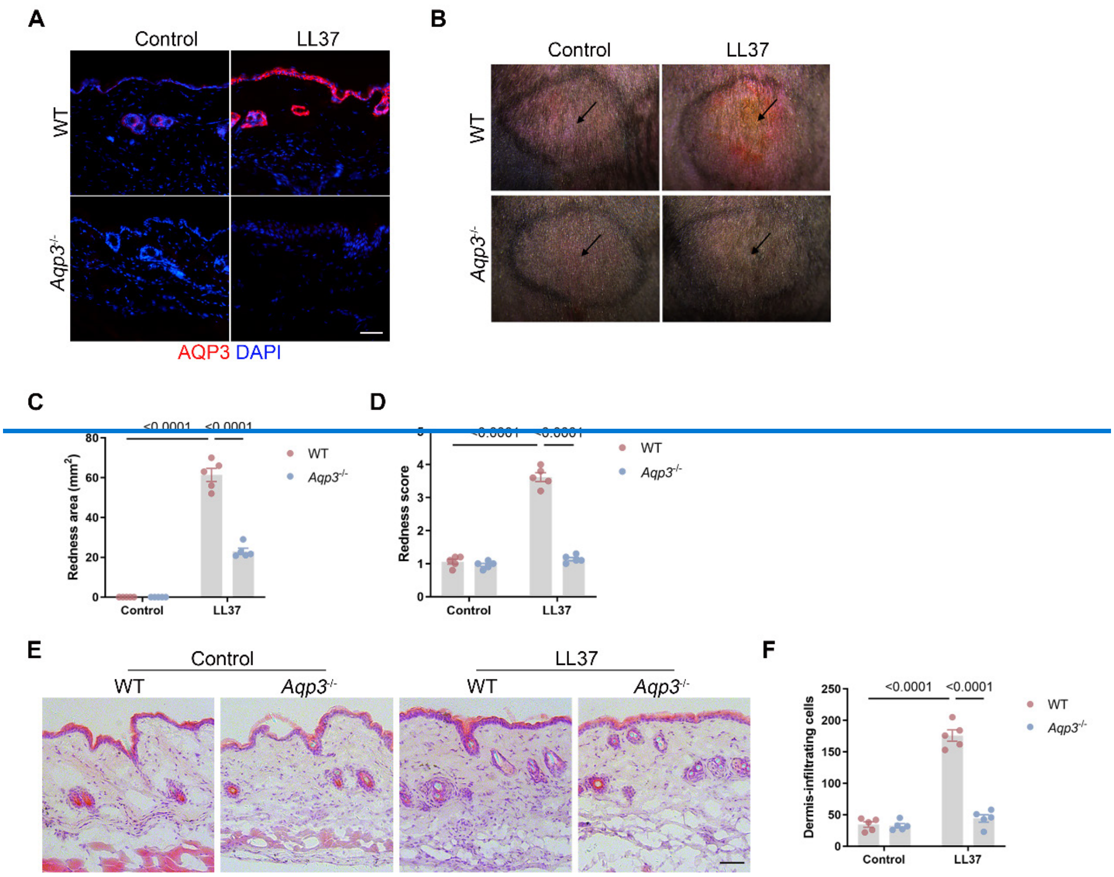

Figure S2. AQP3 deletion alleviates rosacea-like features in mice

(A) Immunostaining of AQP3 in skin sections from WT and *Aqp3*$^{-/-}$ mice induced by LL37. Scale bar: 2mm.

(B) The back skins of WT and *Aqp3*$^{-/-}$ mice in C57 background were intradermally injected with LL37(n=5/group). Images were taken 48 hr after the first LL37 injection.

(C) The severity of the rosacea-like phenotype was evaluated on account of the redness area.

(D) The severity of the rosacea-like phenotype was evaluated on account of the redness score.

(E) HE staining of lesional skin sections from WT and *Aqp3*$^{-/-}$ mice injected with LL37(n=5/group). Scale bar: 50μm.

(F) Quantitative result of H&E staining for dermal cellular infiltrates is shown. Data represent the mean ± SEM.One-way ANOVA with Bonferroni's post hoc

test was used.

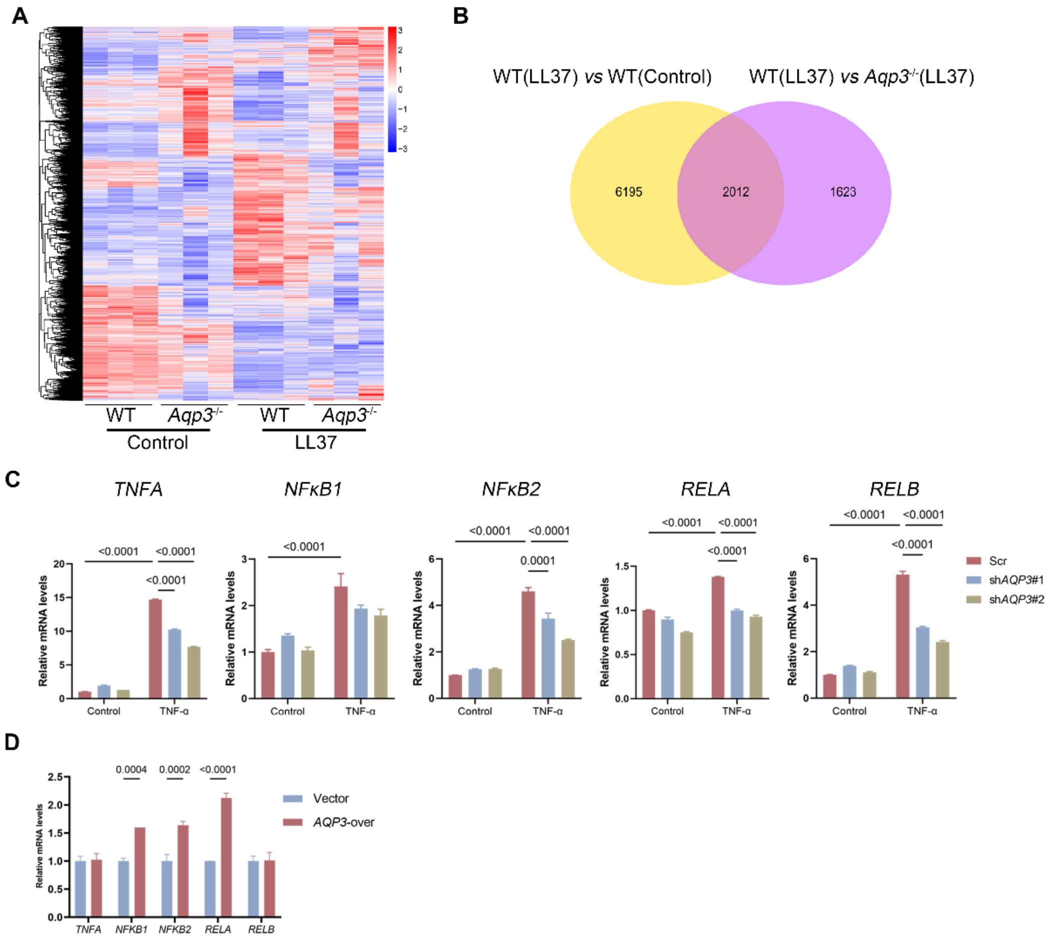

Figure S3. AQP3 positively regulates NF-κB activation in keratinocyte.

(A) Heatmap of differentially regulated genes in skin lesions from LL37-injected WT and *Aqp3*$^{-/-}$ mice determined by RNA-sequencing (n = 3 independent biological samples for each group). Blue color denotes low FPKM expression; red, high FPKM expression.

(B) Venn diagram showing the overlap DEGs between the two comparisons (WT (LL37) vs WT (Control), and WT (LL37) vs *Aqp3*$^{-/-}$ (LL37)).

(C) The mRNA expression levels of TNFα and NF-κB family of transcription factors (NFKB1, NFKB2, RELA, and RELB) in keratinocytes transfected with scrambled or AQP3 shRNA.

(D) The mRNA levels of NF-κB family of transcription factor and target genes in keratinocytes transfected with AQP3 cDNA or control vector.

Data represent the mean ± SEM. Two-tailed unpaired Student's t-test (**C**) or

1-way ANOVA with Bonferroni's post hoc test (**D and E**) was used.

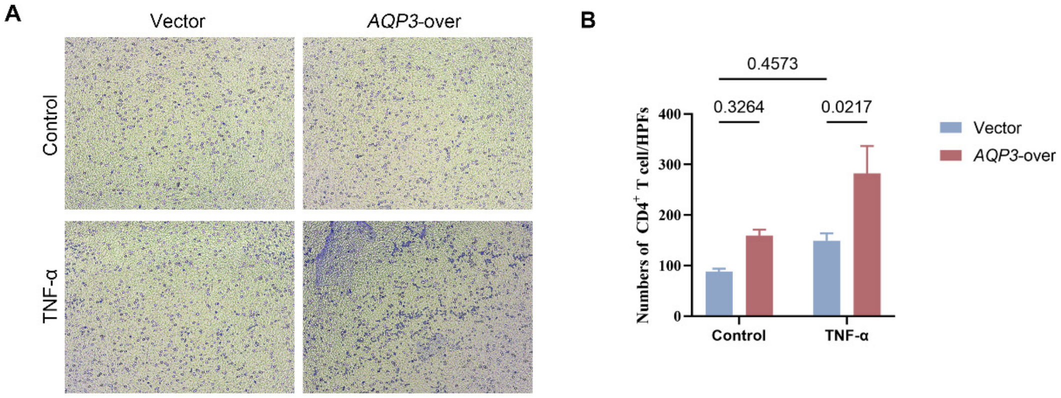

Figure S4. The enhanced expression of AQP3 in keratinocyte promotes CD4^{+} T cell chemotaxis in the presence of TNF-α.

(A) Representative images of migrated T cells cocultured with AQP3-overexpressing or vector HaCaT keratinocytes on the undersurface of the transwell.

(B) Quantification of migrated T cells

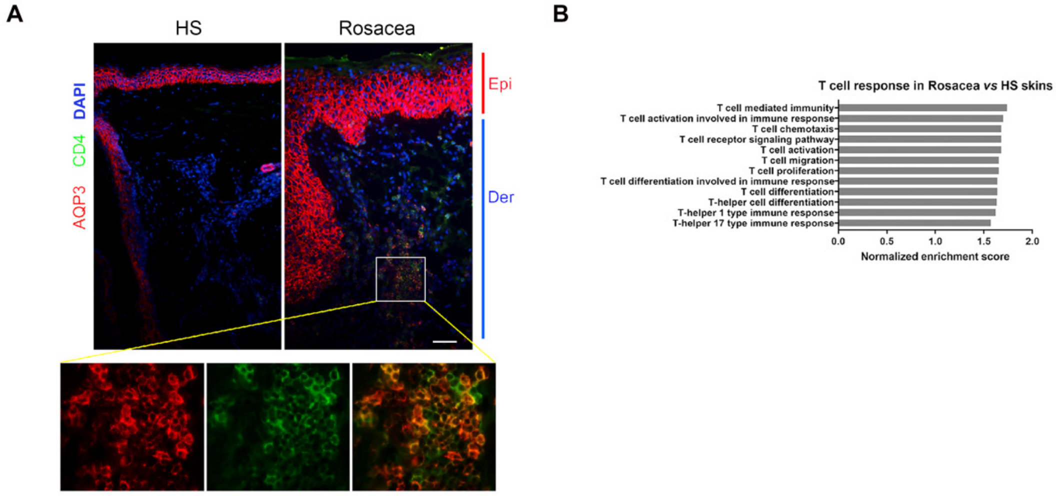

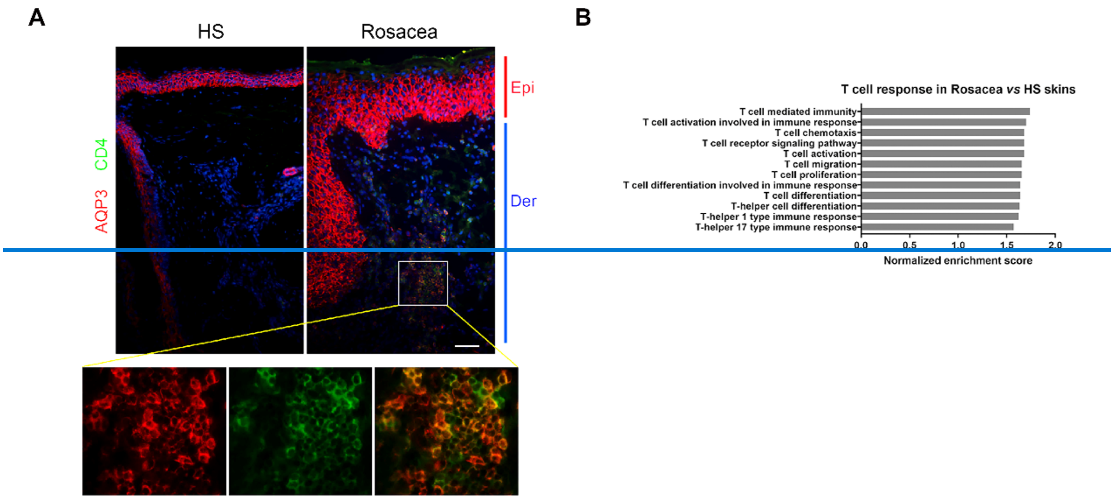

Figure S5. AQP3 is also upregulated in CD4^{+} T cell.

(A) Immunostaining of AQP3 (red), ~~and~~ CD4 (green) and nucleus (blue) in rosacea patients and HS samples. Scale bar: 50 μm.

(B) Top-ranked upregulated KEGG terms related to T cell responses in genes that were differentially regulated between rosacea and HS samples revealed through GSEA.

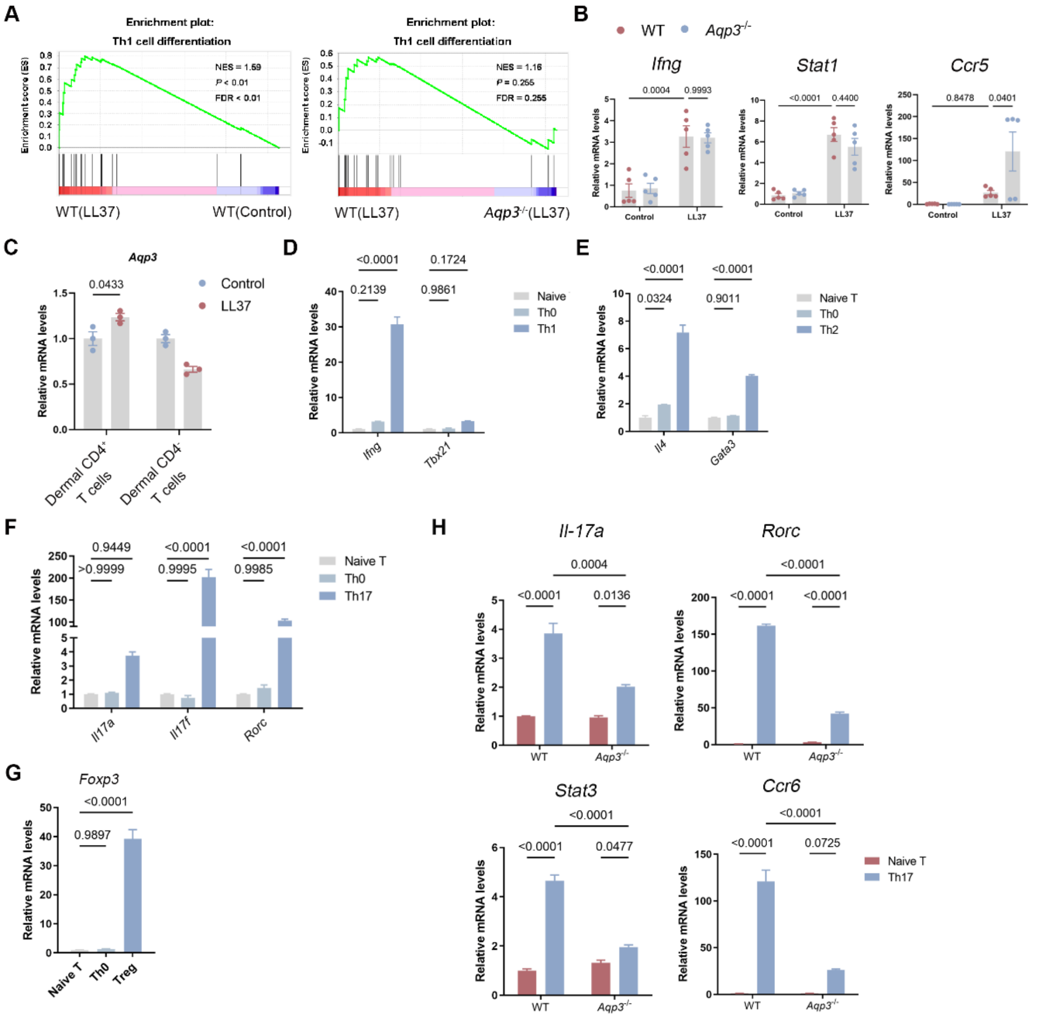

Figure S6. AQP3 regulates Th17-polarization inflammation.

(A) GSEA of Th1 cell differentiation on RNA-sequencing data from the two comparisons (WT (LL37) vs WT (Control), and WT (LL37) vs *Aqp3*$^{-/-}$ (LL37)). Significance was calculated by permutation test.

(B) The mRNA expression levels of Th1 polarization-related genes (*Ifng*, *Stat1* and *Ccr5*) in mice skin lesions.

(C) Expression of *Aqp3* in dermal CD4^{-} T cells of skin samples from LL37-treated mice.

(D-G) I*n vitro* differentiation of mouse naïve CD4^{+} T cells. The mRNA levels of corresponding specific cytokines and transcription factors in naïve CD4+ T cells, Th0 cells and polarized Th cells were detected by RT-qPCR.

(**H**) The mRNA expression of Th17 polarization-related genes in WT or *Aqp3*$^{-/-}$

naïve CD4⁺ T or Th17 cells. Data represent the mean ± SEM. Two-tailed unpaired Student's t-test or 1-way ANOVA with Bonferroni's post hoc test was used.

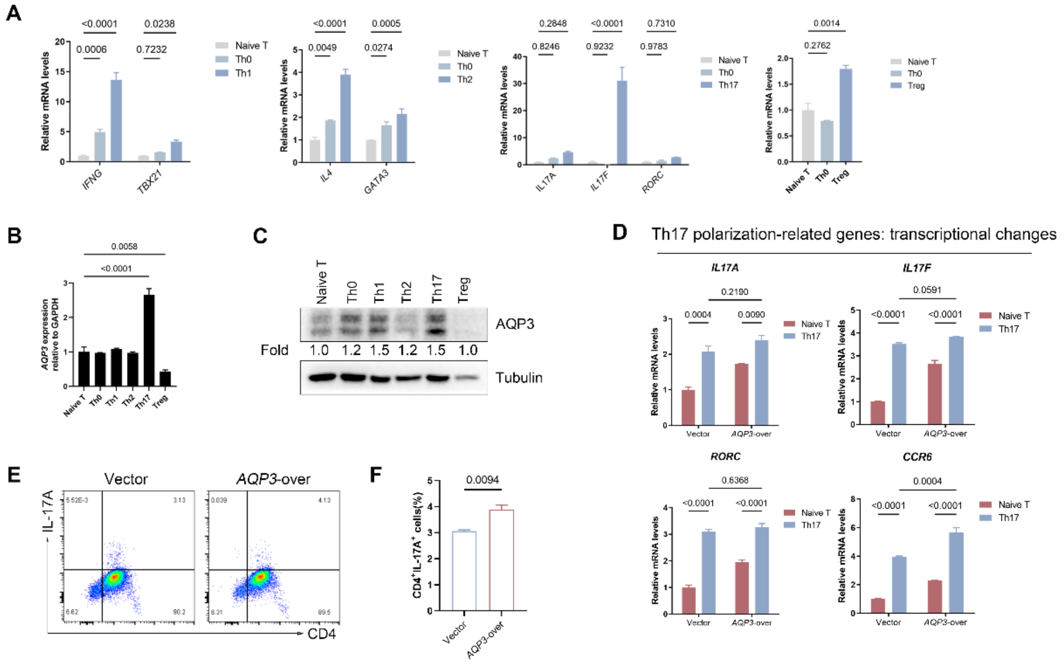

Figure S7. AQP3 involves in human Th17 cell differentiation.

(A) In vitro differentiation of human naïve CD4⁺ T cells. The mRNA levels of corresponding specific cytokines and transcription factors in naïve CD4⁺ T cells, Th0 cells and polarized Th cells were detected by RT-qPCR.

(B) The mRNA expression levels of AQP3 in freshly isolated CD4 T cells (naive) and polyclonally activated CD4⁺ T cells (Th0) and Th1, Th2, Th17, and iTreg cells.

(C) Immunoblotting of AQP3 in freshly isolated CD4⁺ T cells (naive) and polyclonally activated CD4⁺ T cells (Th0) and Th1, Th2, Th17, and iTreg cells. Tubulin was used as a loading control.

(D) Human naive CD4⁺ T cells isolated from PBMC and then were infected with Control vector or AQP3 cDNA lentivirus under the differentiation condition of Th17 cells. The percentage of Th17 cells was detected by flow cytometry.

(E) Statistical analysis data of the percentage of Th17 cells in (D). All results

are representative of at least 3 independent experiments. Data represent the mean ± SEM. Two-tailed unpaired Student's t-test (E) or 1-way ANOVA with Bonferroni's post hoc test was used.

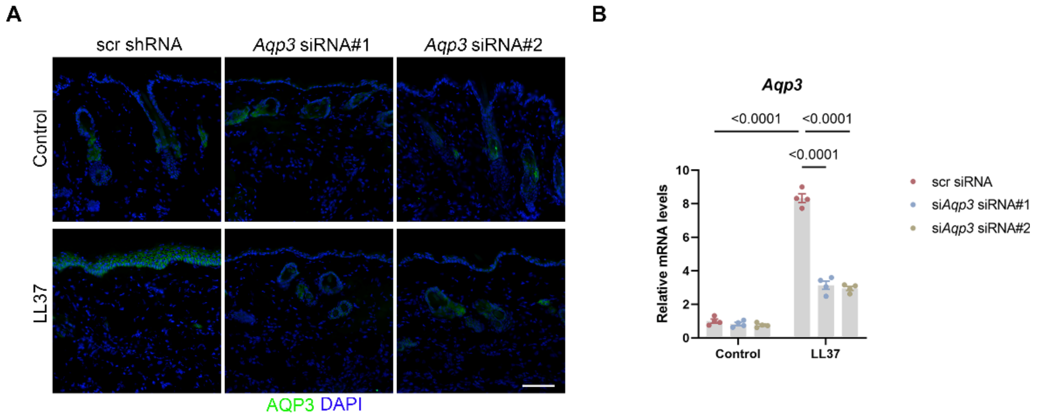

Figure S8. Validation of the knockdown effect of Aqp3 siRNA in the skin.

(A) Immunostaining of Aqp3 in skin lesions from Scr and Aqp3 siRNAs-administered mice injected with LL37 or control vehicle (n=4/group). DAPI staining (blue) indicates nuclear localization. Scale bar: 50 μm.

(B) The mRNA expression of Aqp3 in in skin lesions from Scr and Aqp3 siRNAs-administered mice injected with LL37 or control vehicle (n=4/group). Data represent the mean ± SEM. 1-way ANOVA with Bonferroni's post hoc test was used.

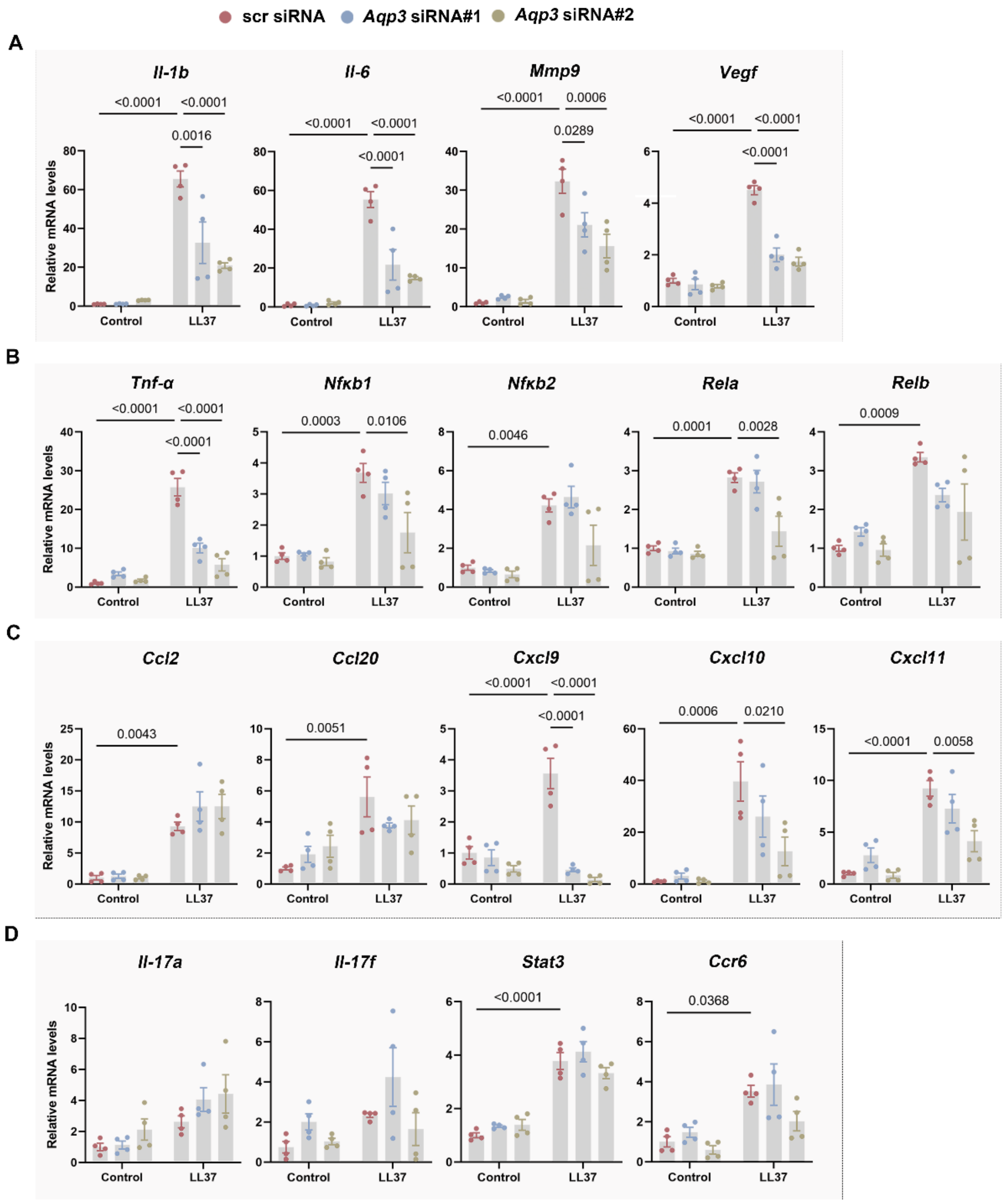

Figure S9. Deficiency of Aqp3 suppresses rosacea development

(A) The mRNA expression of disease-characteristic genes in in skin lesions from Scr and Aqp3 siRNAs-administered mice injected with LL37 or control vehicle (n=4/group).

(B) The mRNA expression of NF-κB family of transcription factors (Nfkb1, Nfkb2, Rela and Relb) in skin lesions from Scr and Aqp3 siRNAs-administered mice injected with LL37 or control vehicle (n=4/group).

(C) The mRNA expression of chemokines in skin lesions from Scr and Aqp3

siRNAs-administered mice injected with LL37 or control vehicle (n=4/group)

(D) The mRNA expression of Th17 polarization-related genes (Il17a, Il17f, Stat3 and Ccr6) in skin lesions from Scr and Aqp3 siRNAs-administered mice injected with LL37 or control vehicle (n=4/group)

Data represent the mean ± SEM. 1-way ANOVA with Bonferroni's post hoc test was used.

Supplemental Table 1. Primer sequences

qPCR primers	
Human-*GAPDH*-F	TGTTGCCATCAATGACCCCTT
Human-*GAPDH*-R	CTCCACGACGTACTCAGCG
Human-*AQP3*-F	TCTTTGACCAGTTCATAGGCAC
Human-*AQP3*-R	GGCAGGGTTGACGGCATAG
Human-*CXCL8*-F	TTTTGCCAAGGAGTGCTAAAGA
Human-*CXCL8*-R	AACCCTCTGCACCCAGTTTTC
Human-*CXCL9*-F	CCAGTAGTGAGAAAGGGTCGC
Human-*CXCL9*-R	AGGGCTTGGGGCAAATTGTT
Human-*CXCL10*-F	GTGGCATTCAAGGAGTACCTC
Human-*CXCL10*-R	TGATGGCCTTCGATTCTGGATT
Human-*CXCL11*-F	GACGCTGTCTTTGCATAGGC
Human-*CXCL11*-R	GGATTTAGGCATCGTTGTCCTTT
Human-*CCL1*-F	CTCATTTGCGGAGCAAGAGAT
Human-*CCL1*-R	GCCTCTGAACCCATCCAACTG
Human-*CCL2*-F	CAGCCAGATGCAATCAATGCC
Human-*CCL2*-R	TGGAATCCTGAACCCACTTCT
Human-*CCL3*-F	AGTTCTCTGCATCACTTGCTG
Human-*CCL3*-R	CGGCTTCGCTTGGTTAGGAA
Human-*CCL20*-F	TGCTGTACCAAGAGTTTGCTC
Human-*CCL20*-R	CGCACACAGACAACTTTTTCTTT
Human-*CCL27*-F	GCAGCATTCCTACTGCCAC
Human-*CCL27*-R	AGGTGAAGCACGAAAGCCTG

Human-*IL1B*-F	TTCGACACATGGGATAACGAGG
Human-*IL1B*-R	TTTTTGCTGTGAGTCCCGGAG
Human-*IL6*-F	CCTGAACCTTCCAAAGATGGC
Human-*IL6*-R	TTCACCAGGCAAGTCTCCTCA
Human-*TNF-α*-F	CCTCTCTCTAATCAGCCCTCTG
Human-*TNF-α*-R	GAGGACCTGGGAGTAGATGAG
Human-*NFKB1*-F	AACAGAGAGGATTTCGTTTCCG
Human- *NFKB1*-R	TTTGACCTGAGGGTAAGACTTCT
Human-*NFKB2*-F	ATGGAGAGTTGCTACAACCCA
Human- *NFKB2*-R	CTGTTCCACGATCACCAGGTA
Human-*RELA*-F	ATGTGGAGATCATTGAGCAGC
Human-*RELA*-R	CCTGGTCCTGTGTAGCCATT
Human-*RELB*-F	CCATTGAGCGGAAGATTCAACT
Human-*RELB*-R	CTGCTGGTCCCGATATGAGG
Human-*IFNG*-F	CATCCAAAAGAGTGTGGAGACA
Human-*IFNG*-R	TGCTTTGCGTTGGACATTCAAG
Human-*TBX21*-F	CAGGGACGGCGGATGTTCC
Human-*TBX21*-R	TCCACACTGCACCCACTTGC
Human-*IL4*-F	GGTCACAGGAGAAGGGACGCC
Human-*IL4*-R	TGCGAAGCACCTTGGAAGCCC
Human-*GATA3*-F	ACAGAACCGGCCCCTCATTAA
Human-*GATA3*-R	TGGTCTGACAGTTCGCACAGGA
Human-*IL17A*-F	ATTACTACAACCGATCCACCTC
Human-*IL17A*-R	TGGTAGTCCACGTTCCCAT
Human-*IL17F*-F	AGTAAGCCACCAGCGCAACATG
Human-*IL17F*-R	CTCAGAAAGGCAAGCCCCAATA
Human-*RORC*-F	CTGGGCATGTCCCGAGATG
Human-*RORC*-R	GAGGGGTCTTGACCACTGG
Human-*FOXP3*-F	GTGGCCCGGATGTGAGAAG
Human-*FOXP3*-R	GGAGCCCTTGTCGGATGATG
Mouse-*Gapdh*-F	AGGTCGGTGTGAACGGATTTG
Mouse-*Gapdh*-R	TGTAGACCATGTAGTTGAGGTCA
Mouse-*Aqp3*-F	CCTTGGCATCTTGGTGGCT

Mouse-*Aqp3*-R	AGGAAGCACATTGCGAAGGT
Mouse-*Tnfα*-F	CTGAACTTCGGGGTGATCGG
Mouse-*Tnfα*-R	GGCTTGTCACTCGAATTTTGAGA
Mouse-*Il1b*-F	GCAACTGTTCCTGAACTCAACT
Mouse-*Il1b*-R	ATCTTTTGGGGTCCGTCAACT
Mouse-*Il6*-F	TAGTCCTTCCTACCCCAATTTCC
Mouse-*Il6*-R	TTGGTCCTTAGCCACTCCTTC
Mouse-*Mmp9*-F	CTGGACAGCCAGACACTAAAG
Mouse-*Mmp9*-R	CTCGCGGCAAGTCTTCAGAG
Mouse-*Nfkb1*-F	ATGGCAGACGATGATCCCTAC
Mouse-*Nfkb1*-R	TGTTGACAGTGGTATTTCTGGTG
Mouse-*Nfkb2*-F	GGCCGGAAGACCTATCCTACT
Mouse-*Nfkb2*-R	CTACAGACACAGCGCACACT
Mouse-*Rela*-F	AGGCTTCTGGGCCTTATGTG
Mouse-*Rela*-R	TGCTTCTCTCGCCAGGAATAC
Mouse-*Relb*-F	CCGTACCTGGTCATCACAGAG
Mouse-*Relb*-R	CAGTCTCGAAGCTCGATGGC
Mouse-*Cxcl9*-F	GGAGTTCGAGGAACCCTAGTG
Mouse-*Cxcl9*-R	GGGATTTGTAGTGGATCGTGC
Mouse-*Cxcl10*-F	CCAAGTGCTGCCGTCATTTTC
Mouse-*Cxcl10*-R	GGCTCGCAGGGATGATTTCAA
Mouse-*Cxcl11*-F	GGCTTCCTTATGTTCAAACAGGG
Mouse-*Cxcl11*-R	GCCGTTACTCGGGTAAATTACA
Mouse-*Cxcl12*-F	TGCATCAGTGACGGTAAACCA
Mouse-*Cxcl12*-R	TTCTTCAGCCGTGCAACAATC
Mouse-*Cx3cl1*-F	ACGAAATGCGAAATCATGTGC
Mouse-*Cx3cl1*-R	CTGTGTCGTCTCCAGGACAA
Mouse-*Ccl2*-F	TTAAAAACCTGGATCGGAACCAA
Mouse-*Ccl2*-R	GCATTAGCTTCAGATTTACGGGT
Mouse-*Ccl5*-F	GCTGCTTTGCCTACCTCTCC
Mouse-*Ccl5*-R	TCGAGTGACAAACACGACTGC
Mouse-*Ccl20*-F	GCCTCTCGTACATACAGACGC
Mouse-*Ccl20*-R	CCAGTTCTGCTTTGGATCAGC

Mouse-*Vegf*-F	TATTCAGCGGACTCACCAGC
Mouse-*Vegf*-R	AACCAACCTCCTCAAACCGT
Mouse-*Il17a*-F	ATGCTGTTGCTGCTGCTGAG
Mouse-*Il17a*-R	GGAAGTCCTTGGCCTCAGTG
Mouse-*Il17f*-F	GGAGGTAGCAGCTCGGAAGA
Mouse-*Il17f*-R	GGAGCGGTTCTGGAATTCAC
Mouse-*Stat3*-F	CAATACCATTGACCTGCCGAT
Mouse-*Stat3*-R	GAGCGACTCAAACTGCCCT
Mouse-*Ccr6*-F	CCTGGGCAACATTATGGTGGT
Mouse-*Ccr6*-R	CAGAACGGTAGGGTGAGGACA
Mouse-*Rorc*-F	GGACAGGGAGCCAAGTTCTCA
Mouse-*Rorc*-R	CACAGGTGATAACCCCGTAGTGG
Mouse-*Ifng*-F	AGACAATCAGGCCATCAGCA
Mouse-*Ifng*-R	CAACAGCTGGTGGACCACTC
Mouse-*Tbx21*-F	CAACAACCCCTTTGCCAAAG
Mouse-*Tbx21*-R	TCCCCCAAGCAGTTGACAGT
Mouse-*Stat1*-F	CGGAGTCGGAGGCCCTAAT
Mouse-*Stat1*-R	ACAGCAGGTGCTTCTTAATGAG
Mouse-*Ccr5*-F	ATGGATTTTCAAGGGTCAGTTCC
Mouse-*Ccr5*-R	CTGAGCCGCAATTTGTTTCAC
Mouse-*Il4*-F	GGTCTCAACCCCCAGCTAGT
Mouse-*Il4*-R	GCCGATGATCTCTCTCAAGTGAT
Mouse-*Gata3*-F	AGAACCGGCCCCTTATGAA
Mouse-*Gata3*-R	AGTTCGCGCAGGATGTCC
